# Musculoskeletal modelling of the Nile crocodile (*Crocodylus niloticus*) hindlimb: Effects of limb posture on leverage during terrestrial locomotion

**DOI:** 10.1111/joa.13431

**Published:** 2021-03-23

**Authors:** Ashleigh L. A. Wiseman, Peter J. Bishop, Oliver E. Demuth, Andrew R. Cuff, Krijn B. Michel, John R. Hutchinson

**Affiliations:** ^1^ Structure and Motion Laboratory Comparative Biomedical Sciences Royal Veterinary College Hatfield UK; ^2^ Geosciences Program Queensland Museum Brisbane Qld Australia; ^3^ Department of Organismic and Evolutionary Biology Harvard University Cambridge USA; ^4^ Department of Earth Sciences University of Cambridge Cambridge UK; ^5^ Hull York Medical School University of York York UK

**Keywords:** anatomy, archosaur, biomechanics, locomotion, musculoskeletal modelling, posture, XROMM

## Abstract

We developed a three‐dimensional, computational biomechanical model of a juvenile Nile crocodile (*Crocodylus niloticus*) pelvis and hindlimb, composed of 47 pelvic limb muscles, to investigate muscle function. We tested whether crocodiles, which are known to use a variety of limb postures during movement, use limb orientations (joint angles) that optimise the moment arms (leverages) or moment‐generating capacities of their muscles during different limb postures ranging from a high walk to a sprawling motion. We also describe the three‐dimensional (3D) kinematics of the crocodylian hindlimb during terrestrial locomotion across an instrumented walkway and a treadmill captured via X‐ray Reconstruction of Moving Morphology (biplanar fluoroscopy; ‘XROMM’). We reconstructed the 3D positions and orientations of each of the hindlimb bones and used dissection data for muscle lines of action to reconstruct a focal, subject‐specific 3D musculoskeletal model. Motion data for different styles of walking (a high, crouched, bended and two types of sprawling motion) were fed into the 3D model to identify whether any joints adopted near‐optimal poses for leverage across each of the behaviours. We found that (1) the hip adductors and knee extensors had their largest leverages during sprawling postures and (2) more erect postures typically involved greater peak moment arms about the hip (flexion‐extension), knee (flexion) and metatarsophalangeal (flexion) joints. The results did not fully support the hypothesis that optimal poses are present during different locomotory behaviours because the peak capacities were not always reached around mid‐stance phase. Furthermore, we obtained few clear trends for isometric moment‐generating capacities. Therefore, perhaps peak muscular leverage in Nile crocodiles is instead reached either in early/late stance or possibly during swing phase or other locomotory behaviours that were not studied here, such as non‐terrestrial movement. Alternatively, our findings could reflect a trade‐off between having to execute different postures, meaning that hindlimb muscle leverage is not optimised for any singular posture or behaviour. Our model, however, provides a comprehensive set of 3D estimates of muscle actions in extant crocodiles which can form a basis for investigating muscle function in extinct archosaurs.

## Graphical abstract

We created an open‐source computational 3D biomechanical model of the Nile crocodile hindlimb containing 47 digitally dissected muscles, permitting, for the first time, a comprehensive investigation into muscular leverage in each of the locomotory behaviours/postures used by crocodiles. Here we integrate data from three‐dimensional anatomy, 3D locomotor kinematics in vivo and 3D biomechanical modelling to quantify how Nile crocodiles move. We found that our results did not fully support the hypothesis that biomechanically optimal poses (in terms of leverage) are adopted during different locomotory behaviours in Nile crocodiles. Therefore, we infer that the wide range of locomotor behaviours used by crocodylians on land prohibits simple optimisation of muscle leverage to any single particular behaviour.

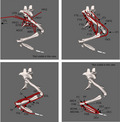

## 1 INTRODUCTION

Mathematical‐computational modelling approaches that can accurately estimate biological and musculoskeletal functions have offered unique insights into biomechanical function in the terrestrial locomotion of extant vertebrates (e.g. Cox et al., 2019; Delp et al., 1990; De Groote et al., 2009; De Groote et al., 2016; Hutchinson, 2012; O’Neill et al., 2013; Hutchinson et al., 2015; Regnault & Pierce, 2018; Poncery et al., 2019; Seth et al., 2018), which can, in turn, provide the basis for modelling and simulating extinct animal locomotion (e.g. Allen et al., 2021; Bates et al., 2010; Bates et al., 2015; Bishop et al., 2020b; Hutchinson et al., 2005; Nyakatura & Demuth, 2019; Sellers & Manning, 2007; Sellers et al., 2009;). Ideally, modelling musculoskeletal function in extinct forms first requires modern forms to be explored, modelled and analysed for validation purposes. Approaches that integrate experimental data with those from informed dissection data of extant forms have pioneered musculoskeletal locomotory modelling for both extant and extinct animals (e.g. Bates & Schachner, 2012; Bishop et al., 2020b; Cuff et al., 2019; Holowka & O’Neill, 2013; Modenese & Kohout, 2020; Otero et al., 2017; Rankin et al., 2016; Sellers et al., 2013; Wang et al., 2004; Wang et al., 2012).

One such group of animals that is of great interest for studies of locomotor biomechanics is Archosauria, the group that includes crocodylians (Crocodylia), birds (Aves) and a host of related extinct forms (e.g. non‐avian dinosaurs and pterosaurs). The lineage Archosauria has a deep and complex evolutionary history (e.g. Nesbitt et al., 2013; Sereno, 1991; Sues, 2019): it originated ~250 million years ago (Ma), diversified considerably on land in the Triassic period, experienced a mass extinction around the Triassic–Jurassic boundary and then diversified again in the Jurassic‐Cretaceous before suffering another mass extinction at the end‐Cretaceous; leaving but two groups still surviving today, as the Crocodylia and Aves. Within the Mesozoic (especially Triassic; 252–201 Ma) there existed numerous forms of archosaur, covering a wide array of skeletal morphologies, which likely correspond to wide differences in locomotor behaviours (Charig, 1972; Demuth et al., 2020; Hutchinson, 2006; Iijima & Kobayashi, 2014; Mallison, 2010; Padian et al., 2010; Parrish, 1986; Sereno, 1991). Investigating the diversification of these musculoskeletal forms and estimating locomotor performance of fossil archosaurs has been a popular scientific subject for about a century (e.g. Allen et al., 2021; Bates & Schachner, 2012; Bishop et al., 2020a; Bonaparte, 1984; Gatesy, 1990; Gauthier et al., 2011; Grinham et al., 2019; Hutchinson, 2006; Meers, 2003; Romer, 1923; Tsai et al., 2020), but has advanced considerably in recent years with the inception and improvement of software/hardware designed to quantify animal mechanics, thus providing detailed biomechanical models that were impossible 30 years ago. In addition to the computational modelling and simulation tools noted above, new advances have permitted researchers to see beneath the skin of animals to visualise and accurately quantify musculoskeletal function during a variety of motions (X‐ray Reconstruction of Moving Morphology or XROMM; Baier & Gatesy., 2013; Brainerd et al., 2010; Gatesy et al., 2010; Kambic et al., 2014; Tsai et al., 2020). Other methods have permitted the non‐destructive exploration of internal bone architecture, thus permitting insights into bone loading patterns (Bishop et al., 2018b; Bishop et al., 2019; Kivell, 2016; Tsegai et al., 2018). Furthermore, other studies have explored digital dissection, providing detailed and precise visualisation of muscle paths or architecture (Dickinson et al., 2019; Klinkhamer et al., 2017; Kupczik et al., 2015; Modenese & Kohout, 2020).

To better understand locomotion in extinct members of the Archosauria clade, we need to quantify the form and function of comparable living archosaurs. For example, to reliably model the locomotory behaviour of the extinct quadrupedal ‘rauisuchian’ *Batrachotomus* (Gower & Schoch, 2009), it would be valuable to first model the similar living *Crocodylus*. Crocodiles employ a continuum of non‐parasagittal limb postures during terrestrial locomotion, ranging from sprawling to a high walk (Gatesy, 1991). The general kinematics of these gaits have been previously well‐studied (e.g. Blob & Biewener, 2001; Brinkman, 1980; Gatesy, 1991; Parrish, 1986; Reilly & Blob, 2003; Reilly & Ellias, 1998; Reilly et al., 2005; Whitaker & Andrews, 1988), in addition to faster modes of locomotion such as asymmetrical bounding and galloping gaits (Hutchinson et al., 2019; Renous et al., 2002). Previous studies of locomotory behaviour have demonstrated that some smaller animals tend to employ more crouched postures, whereas some larger animals use more upright postures to optimise mechanical advantage (e.g. Full & Ahn, 1995; Gatesy & Biewener, 1991; Hutchinson et al., 2015; Reilly et al., 2007) for supporting their body weight (e.g. Biewener, 1989; Günther et al., 2004). However, a trade‐off here may be that smaller animals with crouched postures employ seemingly sub‐optimal joint angles to use greater ranges of joint movement (Daley & Usherwood, 2010). Adult Crocodylia (various species) range from ~10 kg to ~1000 kg (Britton et al., 2012) in body mass. Although a 10 kg crocodile may seem on the larger side of what we can classify as a small animal, crocodiles are an ideal extant species to study the relationship between limb posture (i.e. differing joint rotation angles/limb orientations) and the effect that posture has on the biomechanical capacity to support and move joints because they uniquely adopt a broad variety of limb orientations (e.g. Gatesy, 1991; Reilly & Ellias, 1998). Specifically, how can a group that can vary so dramatically in body size and postural kinematics support its body weight during locomotion (cf. Cieri et al., 2021; Clemente et al., 2011)? And how do the pelvic and hindlimb muscle functions relate to limb orientation and anti‐gravity support during terrestrial locomotion (e.g. Hutchinson & Gatesy, 2000)?

Here we integrate data from three‐dimensional (3D) anatomy (via contrast‐stained scanning), 3D locomotor kinematics in vivo (via XROMM) and 3D biomechanical modelling (via OpenSim software; opensim.stanford.edu; Delp et al., 2007) to quantify how Nile crocodiles move. Our integrated 3D methodology addresses the following questions: (1) Are the moment arms of limb muscles maximised around mid‐stance (coinciding with presumed peak body weight support), or rather do they peak at early/late stance when external joint moments can be highest (Blob & Biewener, 2001)? Alternatively, are moment arms maximised during extreme limb positions (e.g. Hutchinson et al., 2005), such as in the markedly abducted poses used by crocodiles during sprawling postures, perhaps facilitating greater ranges of joint motion (e.g. Lieber, 1997; McClearn, 1985)? And: (2) Do crocodiles adopt certain limb postures which optimise their capacity to generate maximal isometric muscle moments about each of the hindlimb joints, thus promoting economic force production and potentially minimising required active muscle volumes (Cox et al., 2019; Fujiwara, 2009; Fujiwara et al., 2011; Fujiwara & Hutchinson, 2012; Hutchinson et al., 2015; Lieber & Brown, 1992; Lieber & Shoemaker, 1992)? Here, we have created an open‐source computational 3D biomechanical model of the Nile crocodile hindlimb containing 47 digitally dissected muscles, permitting, for the first time, a comprehensive investigation into muscular leverage in each of the locomotory behaviours/postures used by crocodiles.

## 2 MATERIALS AND METHODS

### 2.1 Study animals

An experimental musculoskeletal model was created using data derived from four female juvenile Nile crocodiles (*Crocodylus niloticus* Laurenti 1768; see Cott, 1961). The crocodiles were donated from La Ferme Aux Crocodiles (Pierrelatte, France). The crocodiles were housed in the Biological Services Unit, Structure and Motion Laboratory at the Royal Veterinary College, UK, maintained with basking (UV A + B heat lamps), seclusion and swimming habitats as well as enrichment. Temperatures were kept at ~27°C daytime/~19°C night‐time (12 hr cycle) and with ~70% humidity. In total, there were 10 crocodiles in the enclosures (although only three were used for this study) and they were kept in groups of 1–5 in walled pens and were fed twice weekly on small vertebrates weighing <10% of their body weight. They were transported to/from the experiments in custom‐built crates which were connected directly with the experimental runway, thus minimising human handling. All experimental protocols were conducted in the Structure and Motion Laboratory of the Royal Veterinary College, via prior approval by the College's Ethics and Welfare Committee (approval number 2016‐0089 N) and under a project licence (P0806ABAD) granted by the Home Office (United Kingdom).

### 2.2 Surgical procedure

**FIGURE 1:**
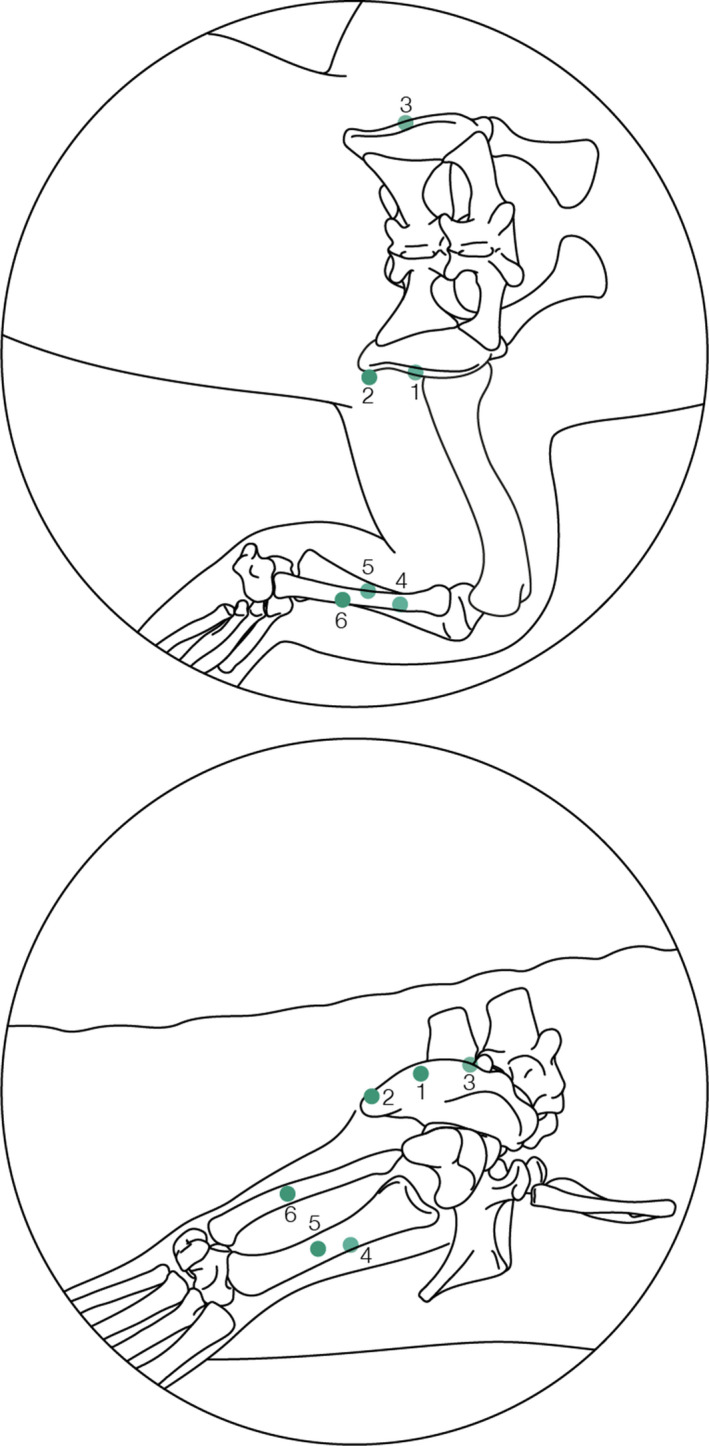
Schematic diagram detailing where each of the markers was surgically implanted in the hindlimb from the dorsal view (a) and right lateral view (b). 1 – right ilium (midpoint); 2 – right caudal ilium; 3 – left ilium (midpoint); 4 – lateral side of the mid‐shaft of the tibia; 5 – lateral side of the mid‐shaft of the right tibia, distal to marker 4; 6 – lateral side of the mid‐shaft of the right fibula

Two crocodiles underwent surgery for bead placement in this study (Table 1). Anaesthesia protocol followed Monticelli et al. (2019) as employed by Cuff et al. (2019) for the same specimens used here. We refer the reader to Cuff et al. (2019) for details on the surgical procedure. Different from Cuff et als. (2019) electromyographic experiments, here six radio‐opaque markers were surgically implanted via six incisions measuring ~1 cm at various points in the pelvis and hindlimb. The first and second markers were inserted into the pelvis on the right cranial and caudal parts of the ilium and the third marker was inserted into the left ilium. The fourth and fifth markers were placed on the lateral right tibia at proximal and distal points along the shaft and the sixth marker was inserted onto the lateral right fibula about midway along with the shaft (Figure 1). These sites were chosen based on surgeons’ judgements weighing surgical accessibility (based on prior cadaver‐based practice) vs. potential impact on surgery duration, animal gait and welfare. Each crocodile had a 7‐day recovery period prior to the commencement of experiments. No subjects showed evidence of locomotor impairments during experiments.

**TABLE 1:** List of *Crocodylus niloticus* specimens used in this study. Two specimens were dissected to provide information on architectural properties, mass properties and skeletal geometry. Two specimens were used in the musculoskeletal modelling and so underwent surgical procedures for bead placement, and one specimen was used as the focal specimen in the final musculoskeletal model, with all other modelling details scaled by femoral length to the final model. DDNC07 was not included in the experiments for this study, but its carcass was used to inform segmental inertial properties

*Specimen ID*	Body mass (kg)	Dissected	CT‐scanned	Underwent surgery	Rigged model	Focal model
DDNC04	3.4	✔	✔	✔	✔	
DDNC06	2.9		✔^a^			✔
DDNC07	4.4	✔	✔			
DDNC10	6.1		✔	✔	✔	

^a^ After euthanasia, this crocodile was iodine‐stained to better visualise muscle paths.

### 2.3 Kinematic data capture

Prior to surgery and experiments, all crocodiles were trained to walk across a straight walkway (measuring 244 cm by 38 cm), a walkway with a bend (measuring 244 cm in total length by 38 cm, with an in‐built bend of 60°) and a motorised treadmill (measuring 100 cm by 40 cm; Starkerhund, Terraglione di Vigodarzere, Italy). Experiments were conducted in the Structure and Motion Lab of the Royal Veterinary College, with a mean temperature of 25°C during experiments and heat lamps used to keep crocodiles warm in between trials. During these experimental trials, the crocodiles were safely moved to the laboratory space and were encouraged to move across a walkway with movement captured via biplanar fluoroscopy using XROMM (Brainerd et al., 2010; Gatesy et al., 2010). Two BV Libra C‐arm systems (Koninklijke Philips N.V., Amsterdam, Netherlands) were used, each composed of a BV 300 generator, F017 tube and BV 300 collimator and intensifier (22.9 cm diameter), with a source‐to‐image distance of 99.5 cm. Photron FASTCAM Mini WX50 high‐speed digital video cameras (Photron, Tokyo, Japan) recorded the trials at 250 frames per second at 2048 × 2048 pixel resolution, with a shutter speed of 1/750 s. The trials were recorded using two GoPro Hero 3+ Silver Edition cameras at 120 Hz, to quantify the speeds of the crocodiles (via a scale object) when moving across the instrumented walkways.

**FIGURE 2:**
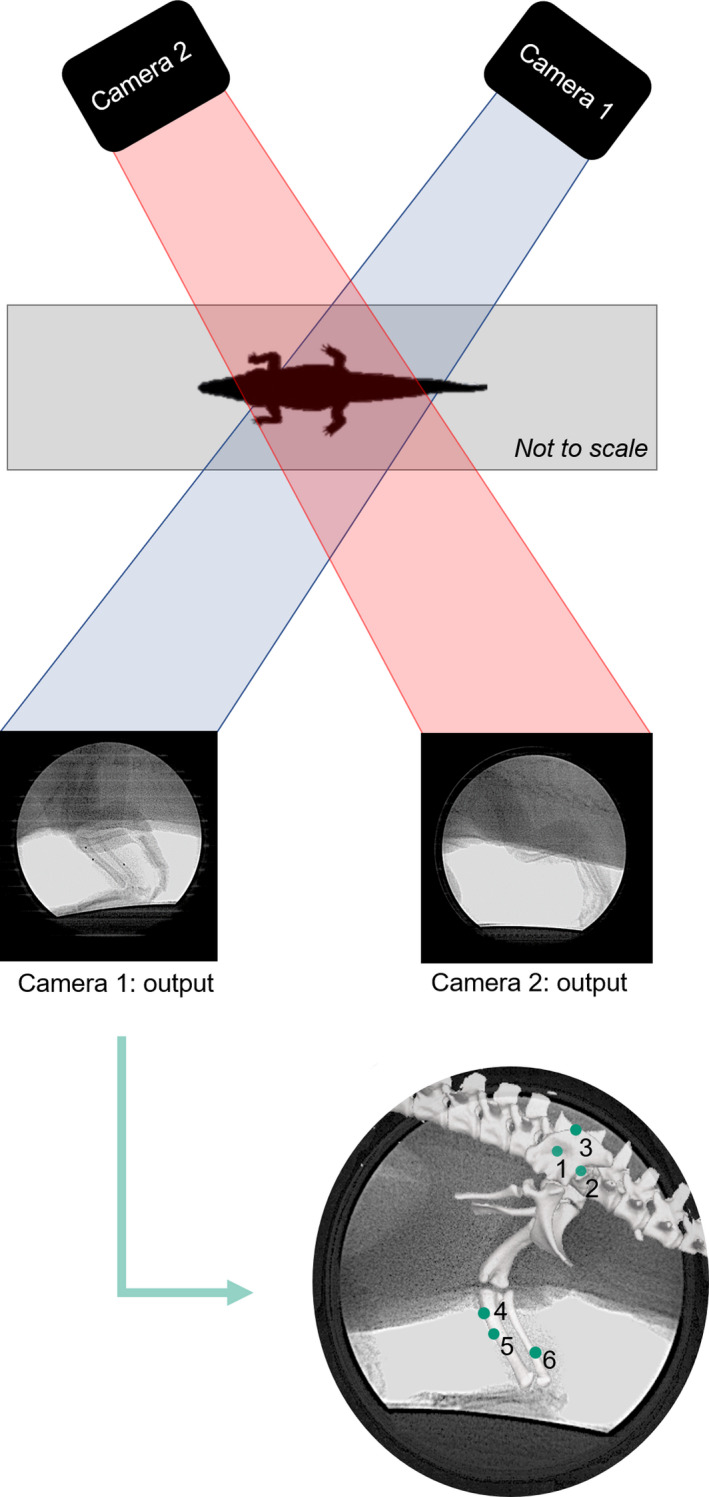
Camera configuration for the trials with an example of each camera output as each specimen walked across either the treadmill or the walkway. These camera outputs were then imported into XMALab (v.1.5.0; Knörlein et al., 2016), where each of the markers was tracked as a 2D point throughout the motion. Refer to Figure 1 caption for marker numbers

The crocodiles walked across the instrumented walkways (straight and bend) at their own chosen speed and limb posture selection (i.e. high or crouched walk). All treadmill trials were set at 0.5 m/s to control for speed and to facilitate the capture of continuous step cycles (Figure 2). This speed was comparable to the trials captured on the regular walkway (~0.4 – 0.62 m/s). A total of 15 trials out of a total of 65 trials (55 trials recorded for DDNC04 and 10 trials recorded for DDNC10) that were recorded were deemed suitable for further analysis. All usable trials for DDNC04 (12 trials) were recorded on the walkways and consist of one step cycle each—no treadmill trials were appropriate from this specimen due to its misbehaviour on the treadmill. Three trials for DDNC10 were recorded on the treadmill for a total of 20 s each, totalling 9 ‐ 13 usable step cycles per trial. However, it was necessary to combine multiple trials together to provide a complete stance phase. Due to the size of the calibrated volume of the XROMM setup, in addition to marker visibility during each of the trials, it was not always possible to fully track all markers throughout the entirety of the gait cycle (for example, one trial captured heel strike and then the hindlimb moved out of the frame towards later stance). Therefore, we adopted a ‘frankensteining’ approach in which multiple trials of similar movement, duty factor and speed were combined together to provide one complete stance cycle (Table 2; see also Bishop et al., in press). Unfortunately, swing phases were not captured for the high walk, crouched walk and bended motions due to a combination of unusable trials and difficulties obtaining a clear view of the hindlimb. Therefore, we only report the stance phase (foot on substrate, as determined by the XROMM video images) results here.

In total, we obtained five different types of continuum locomotory behaviour. Whilst we are not suggesting unique types of locomotory behaviour were observed (except for the bended walk, where each specimen was encouraged to turn around a corner), we have loosely grouped limb postures into behavioural categories based upon the relative distance of the pelvis to the ground during stance (Table 3). We refer to these continua of motions as a high walk, a crouched walk, a bended walk (the crocodiles walked around the 60° bent walkway to encourage a change in direction in travel; see the section above for further details on walkway design and implementation) and two different types of sprawling motion. In one of the types of sprawling behaviours the femur was held in a more protracted position; henceforth this is referred to as ‘sprawl v1’ and the other type with a more retracted femoral position is referred to as ‘sprawl v2’. The sprawling postures can be more easily distinguished by belonging to different trials.

Each specimen was encouraged to locomote across the treadmill for 20 seconds, capturing multiple steps per trial (see above). Some of the treadmill trials were running trots (see duty factors in Table 2). Upon inspection, it was determined that the femur was held in different positions during the movement and thus we chose to compare the moment arms and moments between these two different sprawling types. We selected the one step from each of the sprawling trials which required no ‘frankensteining’. Only these two individual steps from the two sprawling trials are included alongside the other motions in the overall analyses. It was not possible to include additional steps from the treadmill trials due to the limited field of view—often only the forelimb or tail was captured during the trial, not the hindlimb.

**TABLE 2:** Details on trial information, including the number of fragmentary steps captured via XROMM and how many total steps were produced for use in this study via the ‘frankensteining’ method. The bended walking behaviour describes the lateral hindlimb to the bend—that is, the leg on the outside of the turn. ± = standard deviation

		No. of steps	‘Frankensteined’	Duty factor	Trial speed (m/s)
DDNC04	High walk	5	Yes =1 step cycle	0.72 ± 0.01	0.33 ± 0.04 m/s
	Crouched walk	6	Yes =1 step cycle	0.71 ± 0.11	0.58 ± 0.03 m/s
	Bended walk	1	No =1 step cycle	0.75	0.53 m/s
DDNC10	Sprawl v1	13	Yes =5 step cycles	0.47 ± 0.17	0.50 m/s
	Sprawl v2	9	Yes =6 step cycles	0.36 ± 0.14	0.50 m/s

**TABLE 3:** The height of the pelvis ACS origin relative to the ground in each of the continuum motions at 50% of the stance phase. All heights were calculated using trigonometry

Motion	Distance from pelvis to ground (mm)
High walk	106.3 mm
Crouched walk	98.6 mm
Bended walk	104.1 mm
Sprawl v1	70.3 mm^a^
Sprawl v2	69.4 mm^a^

^a^ Whilst the sprawling behaviours indicated that the pelvis to ground distance was comparably similar between these trials (i.e. ~1 mm difference in pelvic height), inspection of the joint ROMs determined that femoral protraction slightly varied between these trials (i.e. femoral retraction was greater in sprawl v2 than sprawl v1—see Table 5 and text) and thus they were separated in this study to determine if any differences existed when femoral protraction differed.

### 2.4 Anatomical digitisation and musculoskeletal construction

After experimentation, all specimens were euthanised following standard ethical procedure using anaesthetic overdose as employed by Cuff et al., (2019). Afterwards, they were immediately frozen at −20°C and maintained until time for dissection, then thawed for 24+ hours at 4°C. Prior to dissection, X‐ray computed tomographic (CT) scanning (Philips Mx8000 IDT16 scanner, 0.80 slice thickness, 0.586 mm pixel resolution, 120 kV, 58 mA, 99 ms exposure at the Queen Mother Hospital, Royal Veterinary College, UK) was used to capture musculoskeletal morphology (all specimens). Furthermore, μCT scanning of DDNC06 (Nikon XTEK XTH 225 ST scanner [Nikon Metrology NV, Leuven, Belgium], 200 kV peak tube voltage, 0.2 mA tube current, 708 ms exposure time, 0.092–0.125 mm isotropic voxel resolution) at the University Museum of Zoology (Cambridge, UK) was used to capture XROMM bead positions, as well as muscle geometry, as follows. Muscle geometry was obtained via first iodine‐staining the hindlimb of DDNC06 (Gignac et al., 2016) (Lugol's solution, 4% iodine with 10% neutral‐buffered formalin, stained for 93 days and then μCT scanning (see details above). We then digitally dissected the muscle bodies, with paths and digital muscle geometries extracted as 3D objects using manual segmentation in Mimics 20.0 (Materialise NV, Leuven, Belgium). Bone geometries and XROMM beads were individually segmented for each specimen to ensure that specific bead placement was incorporated into the 3D models. Muscle lines of actions were generated using the protocol developed by Allen et al., (2017) and Bishop et al., (in press), whereby the lines of action were informed by the 3D volumetric reconstructions of the digitally dissected muscles and a singular line of action was fed through this volume following the centroid of the muscle informed by custom‐written MATLAB code and executed in Rhinoceros 4.0 (Bishop et al., in press). These paths were then imported into OpenSim to create a 3D biomechanical model of the hindlimb.

Two crocodiles (DDNC04 and DDNC07) were dissected following the outline by Hutchinson et al., (2015), with muscle homologies matching those of Romer (1923), Hutchinson (2002) and Hattori and Tsuihiji (2020) (Table 1). DDNC04 provided information on muscle architectural properties, comprising standard measurements (Allen et al., 2014; Martin et al., 2020) of (1) muscle mass (electronic balance ±0.001 g); (2) optimal isometric fascicle length (ℓo) which was assumed equal to dissected resting fibre length (digital callipers, ±0.1 mm; 1–10 measurements/muscle depending on the size and variation of architecture); and (3) pennation θ (protractor, ±5°; 1−5 measurements/muscle under dissecting microscope). These data were used to estimate each muscle's maximum isometric force (henceforth *F*_max_; calculated following Alexander et al., 1979; Lieber & Boakes, 1988; Hutchinson et al., 2015; Allen et al., 2014):

(1) $$F_{\max}=\frac{m\cdot \sigma \cdot \cos\theta }{\rho \cdot l_{O}}$$

where *m* is the muscle mass, σ is the muscle's stress with a value of 300 kN/m^2^ used (Medler, 2002; Michel et al., 2020) and *p* is the tissue density with a density of 1060 kg/m^3^ (Hutchinson et al., 2015; Mendez & Keys, 1960).

Specimen DDNC07 provided data for body segments’ mass properties (not otherwise used herein). DDNC06 was used as the focal specimen in the final musculoskeletal model, with all other modelling details scaled to the focal model (i.e. pelvis translations from each of the experimental trials were scaled by femoral length, although joint rotations were unaltered).

**FIGURE 3:**
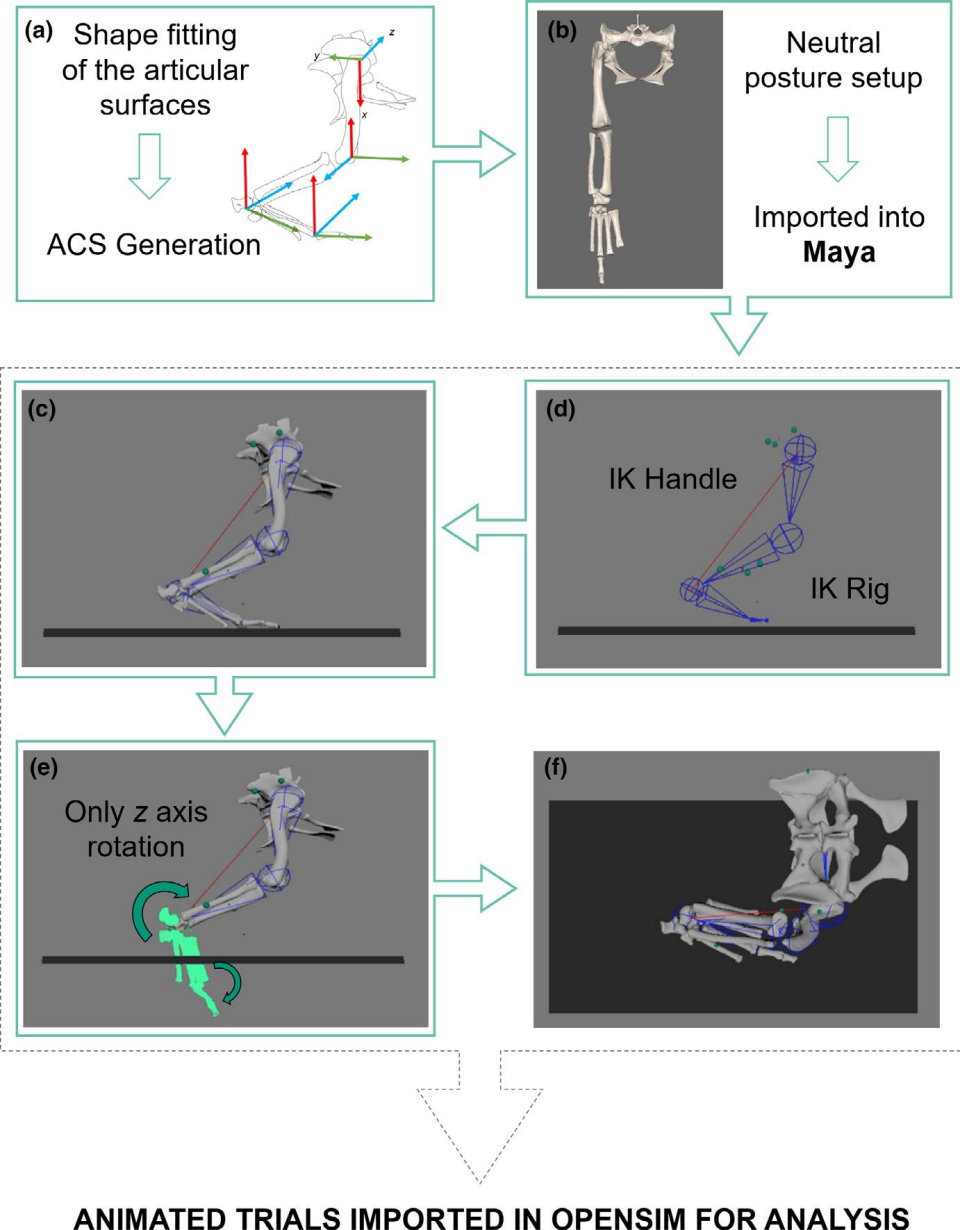
Overview of the inverse kinematic (IK) rigging approach produced in Maya. First, (a) Anatomical Coordinate Systems (ACS) were established following Kambic et al., (2014) and Bishop et al. (in review). The limb was then extended out to create a ‘neutral [or reference] posture’ (Sullivan, 2007) and this limb orientation with all bones in articulation was imported into Maya. (b). The rig was designed and guided by the position of six beads placed in the hindlimb, with the positions of each hindlimb bone constrained via an IK handle (c). The 3D animated points from XMALab were used to animate the beads in Maya, permitting the entire motion to be animated. The pelvis, thigh and shank segments required no rotoscoping as the bones were automatically placed in anatomical position informed by the movement of the beads (d). Only rotoscoping of the ankle and MTP joints (segments shown in light green) was required (e), due to no beads being surgically implanted in the foot bones. The result was a fully animated model with all bones in anatomical positions (f). For details on rig creation, refer to Supplementary Information S1. Only the right leg was modelled in Maya

XROMM data were processed in XMALab (v.1.5.0; Knörlein et al., 2016). The beads were tracked through each of the trials (e.g. Brainerd et al., 2010) and filtered using a low‐pass Butterworth filter of 10 Hz, and then exported as 3D coordinates. Anatomical and joint coordinate systems (ACS/JCS) were established using the shape‐fitting procedure outlined by Bishop et al., (2020), following the protocol designed by Kambic et al., (2014) for birds. Spherical shapes were fitted to each acetabulum and an ellipsoid to the femoral head (c.f. Demuth et al., 2020); cylindrical shapes were fitted to the sacrum, femoral condyles, the ankle (articular surfaces of the astragalus and calcaneum) and the [distal] condyles of the third metatarsal; and planes were fitted to the proximal crus and the proximal articular surface of the third metatarsal (for further details in shape‐fitting refer to Bishop et al., 2020b). JCSs were established for the pelvis, both hip joints, the right knee joint, the right ankle joint and the right third metatarsal joint. All digits were modelled as a singular body for simplicity, meaning that it was only necessary to create an ACS on the midline (the third digit) of the segment. The Z axis was flexion/extension, Y was abduction/adduction and X was long‐axis rotation (Kambic et al., 2014), with the coordinate system as shown in Figure 3a and X‐Y‐Z rotation order in Maya 2019 (Autodesk Inc., San Rafael, USA). The crocodile model was set up in the ‘neutral posture’, with all joints extended (i.e. the limb was vertically straightened into an unnatural pose, with 180‐degree offsets in the flexion‐extension axes) (e.g. Bishop et al., in press; Hutchinson et al., 2005).

The model comprised 12 degrees of freedom (DOFs): three at the hip, one DOF each in the knee, ankle and MTP joints; and six DOFs describing the location and orientation of the pelvis in the global coordinate system. No DOFs were permitted between the fibula and tibia; rather they were modelled as a single unit. Additionally, no translations between joints were permitted, to produce a simplified musculoskeletal model (e.g. Bishop et al., 2020b; Demuth et al., 2020; Hutchinson et al., 2015; Regnault & Pierce, 2018); future innovations of this approach could introduce sliding translations (e.g. Baier & Gatesy, 2013).

We then created a rigged model using an inverse kinematic solver approach (Watt & Watt, 1992) to permit each of the bones to be automatically rotoscoped into position (e.g. Nyakatura & Demuth, 2019) (Figure 3d). This approach used the bead locations as they moved through time to guide the positions of the bones, resulting in the pelvis, thigh and shank segments being locked in anatomical position throughout the motion, by virtue of simplifying assumptions about joint mobility (e.g. number and types of permissible joint movements). Because no markers were placed in the foot and detailed pes kinematics or biomechanics were not a focus of this study, it was only necessary and feasible to rotoscope the metatarsus and digits into anatomical position by changing the angle of the ankle and metatarsophalangeal (MTP) joints whereby only flexion/extension (Z‐axis rotation) of these distal joints was permitted (Figure 3e). The inverse kinematic constrained rig was individually set up for each of the specimens used in this study. After rig creation, any trial belonging to that particular specimen was then fed into the rig for the automatic output of pelvic translations and hindlimb (pelvis, hip and knee) joint rotations. The ankle and MTP joints (Z‐axis rotation, i.e. flexion‐extension) were manually rotoscoped into position (see Gatesy et al., 2010). For comprehensive details on the rig creation, refer to Supplementary Information S1. The result was a set of XROMM‐informed bony motions (Figure 3f) then imported into OpenSim 3.3 with the muscle lines of action, muscle architectural parameters and mass properties for musculoskeletal modelling.

### 2.5 Musculoskeletal modelling

**FIGURE 4:**
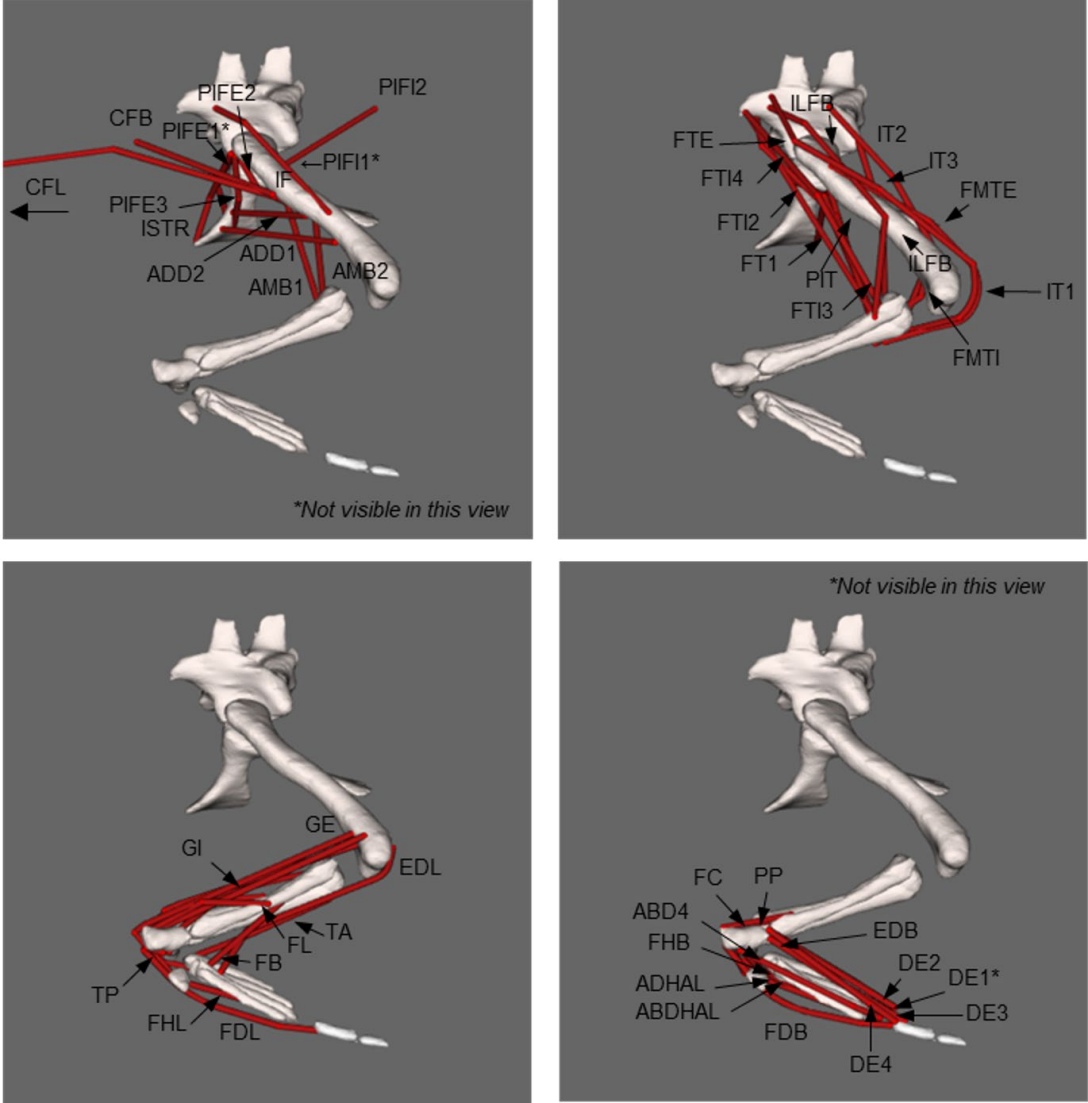
OpenSim model of a *Crocodylus niloticus* right hindlimb, in lateral view. Labelled muscle lines of action are shown in red, loosely grouped according to the major hindlimb joint they act across: (a) the hip, (b) the knee, (c) the ankle and (d) the MTP joints. Refer to Table 4 for muscle abbreviations. Note: The *caudofemoralis longus* (CFL) MTU has been truncated in this figure but continues caudally [Corrections made on 10 April 2021, after first online publication:figure 4 have been updated in this version.]

A 3D musculoskeletal model of the pelvis and right hindlimb was developed for OpenSim 3.3 (Figure 4; Table 4), permitting the computation of muscular moment arms over a range of joint motion (e.g. Delp et al., 2007; Seth et al., 2018; Seth et al., 2011). Each muscle‐tendon unit (MTU) was reconstructed with reference to the muscle lines of action alongside the architectural properties of each muscle, as defined above. In total, this reconstruction produced 47 MTUs in the right hindlimb crossing the hip, knee, ankle and MTP joints, with additional muscles connecting the limb to the body (e.g. the *caudofemoralis longus*). Care was taken to ensure that each MTU did not pass through any other MTU or bone, necessitating the use of ‘via points’ and wrapping surfaces on bone epiphyses (e.g. Arnold et al., 2013; Cox et al., 2019). Some MTU paths were simplified (e.g. the *flexor hallucis longus*) if the muscle was split into multiple heads by combining the muscle heads into one unit (e.g. Hutchinson et al., 2015; Regnault & Pierce, 2018). The creation of wrapping surfaces coupled with the combining of muscle heads might result in muscle function outputs/interpretations being influenced by the researcher's modelling (Brassey et al., 2017; Regnault & Pierce, 2018). Unfortunately, this is a known limitation to musculoskeletal modelling (Hutchinson et al., 2005). Wrapping surfaces and via points produce muscle lines of action that are not straight, but instead can move along a curved path without bony penetration, improving anatomical realism of the model (Jensen & Davey, 1975; Modenese & Kohout, 2020). Model animations are in Supplementary Information 3.

**TABLE 4:** Muscle abbreviations used in this study. In total, 47 muscles were used in the model. See Figure 4 for positions of muscles

Muscle name	Abbreviation	Muscle name	Abbreviation
Caudofemoralis longus	CFL	Ischiotrochantericus	ISTR
Caudofemoralis brevis	CFB	Gastrocnemius internus	GI
Puboischiofemoralis internus 1	PIFI1	Gastrocnemius externus	GE
Puboischiofemoralis internus 2	PIFI2	Tibialis anterior	TA
Puboischiofemoralis externus 1	PIFE1	Tibialis posterior	TP
Puboischiofemoralis externus 2	PIFE2	Extensor digitorum longus	EDL
Puboischiofemoralis externus 3	PIFE3	Flexor digitorum longus	FDL
Adductor 1	ADD1	Extensor hallucis longus	EHL
Adductor 2	ADD2	Fibularis longus	FL
Flexor tibialis externus	FTE	Fibularis brevis	FB
Ambiens 1	AMB1	Flexor hallucis longus	FHL
Ambiens 2	AMB2	Pronator profundus	PP
Iliotibialis 1	IT1	Fibulocalcaneus	FC
Iliotibialis 2	IT2	Abductor (digit) 4	ABD4
Iliotibialis 3	IT3	Flexor hallucis brevis	FHB
Flexor tibialis internus 1	FTI1	Adductor hallucis	ADHAL
Flexor tibialis internus 2	FTI2	Abductor hallucis	ABDHAL
Flexor tibialis internus 3	FTI3	Flexor digitorum brevis	FDB
Flexor tibialis internus 4	FTI4	Extensor digitorum brevis	EDB
Femorotibialis externus	FMTE	Digiti extensor 1	DE1
Femorotibialis internus	FMTI	Digiti extensor 2	DE2
Iliofemoralis	IF	Digiti extensor 3	DE3
Iliofibularis	ILFB	Digiti extensor 4	DE4
Puboischiotibialis	PIT		

### 2.6 Limb muscle biomechanics

To test if muscle moment arms are optimised during different limb postures (e.g. a high walk vs a crouched walk), we calculated 47 MTU moment arms via the ‘virtual work’ method (Delp & Loan, 2000; Pandy, 1999) for each of the postures using the Muscle Analysis tool in OpenSim. To determine if muscle moment arms peaked at adducted limb postures (i.e. the high walk vs the sprawls) or at mid‐stance (i.e. at 50% of stance) of locomotion corresponding to expected peak limb loading, we used the model to calculate the mean moment arm of all adductor, abductor (hip only), extensor or flexor (all joints) muscles across the full range of motion of each joint in adduction/abduction and flexion/extension (set at constant values for mid‐stance in other DOFs), summed these mean moment arms and divided that sum by the summed peak moment arms for each muscle across the same range of motion (as in Hutchinson et al., 2015). We then inspected whether our representative mid‐stance poses during each of the movements included in this study corresponded to peak moment arm values in conjunction with assumed peak limb loading at mid‐stance postures.

To test whether crocodile MTU moment‐generating capacity is optimised to match possible peak moments during each of the different motions (e.g. sprawling motion vs high walk) at 50% of stance which corresponds to probable peak loading, we computed MTU moment‐generating capacities (in Nm) using each muscle's maximal isometric force (F_max_) and moment arm (Hutchinson et al., 2015; O’Neill et al., 2013). We did not account for muscle and tendon force‐length relationships, as per the Hill‐type muscle model (Zajac, 1989), but instead used a simple, static muscle model; intended for comparative purposes as per prior studies. We thus obtained estimates of the variation of maximal isometric moment‐generating capacities throughout different types of locomotory behaviours. The output of the model was represented as a curve of the moment which could be produced by any given muscle around a joint throughout the limb's range of motion (ROM). Each joint's ROM was defined here as the total in vivo ROM observed across each of the trials included in the study. All MTU moments pertaining to a particular action (e.g. hip adduction or knee flexion) were summed, thus assuming that all muscles are maximally activated in their contributions to the total moment.

### 2.7 Monte Carlo simulations

Both moment arms and moments were estimated in this study from small sample sizes. As such, it is probable that our estimates were imprecise, which could then affect our conclusions. To explore the potential error in our moment arm and moment estimates, Monte Carlo simulations were computed in MATLAB using 1000 iterations. For a given muscle, each moment arm/moment was perturbed by a singular, randomly assigned value that produced smooth curves, rather than perturbing each timestep independent of preceding and successive timesteps. For each muscle in a given gait, the original values for each muscle were permitted to deviate up to ±20% from its original value (assuming a random uniform distribution), basing this 20% from Brown et al., (2003), Cox et al., (2019) and Karabulut et al. (2014). In these studies, moment arm data were calculated in different ways and from different estimates, such as estimated values from models versus those from tendon travel data (Cox et al., 2019). Here, we took the average percentage difference (as an absolute value) from the maximum variation in moment arm calculations from these papers and selected the resultant median value of 20%. By the median value of each paper's maximum variation, we adopted a more liberal approach than that of applying the average value from these studies of ~12%, thus conservatively addressing the potential for error to influence our results.

The Monte Carlo simulations were computed in three different ways. First, each muscle's moment arm was permitted to vary up to ±20% from its originally estimated values, after which these values were summed. Second, $F_{\max}$ was varied up to ±20% (henceforth, $F_{max{\;}^{\pm 20\%}}$) after which all individual muscle moment arms were multiplied by the perturbed isometric $F_{max{\;}^{\pm 20\%}}$ values and then summed to provide each DOF’s moment. This method phenomenologically accounts for variation in suboptimal fibre lengths, non‐zero contraction velocities and submaximal activations. Third, each individual moment arm was varied up ±20%, summed and then multiplied by the original $F_{\max}$ values to estimate maximal isometric moments. Each of these methods produced simulated ‘error margins’ for both the moment arms and moments, in which the error margins are considered as an envelope approach. The original data may deviate within said envelope if our original sample size had been greater.

## 3 RESULTS

Our data acquired from a 3D, subject‐specific musculoskeletal model of a crocodile addressed each of our main questions of this study by quantifying muscle moment arms and MTU moment‐generating capacities. Supplementary Information S3 shows the model animated according to each of the five locomotory behaviours included in this study. Here, we present the 3D kinematic data on the ROM of limb joints, followed by summed muscle moment arms, then summed MTU moments.

### 3.1 Joint ranges of motion (ROM)

We report the full ROM used in each of the movements recorded from two experimental Nile crocodiles. Table 5 summarises the hip, knee, ankle and MTP joint ROMs for each of the DOFs across each of the conditions (high walk, crouched walk, both sprawls and the bended walk). Postural changes in the hindlimb can be described according to the ROM of each DOF. A sprawling movement required greater movement around the hip *Z*‐axis (flexion and extension; −69⁰ to 37⁰) than that of a high walk (−19⁰ to −32⁰). As expected, we found that the crouched walk has ROMs intermediate between the high walk and sprawls (Table 5). Although we found discrepancies in all DOFs between the high walk and crouched walk, the greatest discrepancy in joint orientations was hip long axis rotation (LAR), suggesting that this joint motion was the primary DOF facilitating changes in posture. Each of these locomotory behaviours from a continuum of movements separated by changes in pelvic height is shown in Supplementary Information 3.

**TABLE 5:** Mean ranges of motion (ROM) about each of the joint axes for the six DOFs in the hindlimb model. ROMs refer to the maximum and minimum rotations of each motion during the stance phase only. The full ROM of each DOF in these different limb orientations is also reported

	Steps (n)	Hip			Knee	Ankle	MTP
		*Flex‐Ext (Z)*	*Abd‐Ad (Y)*	*LAR (X)*	*Flex‐Ext*	*Flex‐Ext*	*Flex‐Ext*
High walk	1	−19⁰ to −32⁰	27⁰ to 38⁰	−19⁰ to −2⁰	−122⁰ to −69⁰	−40⁰ to −4⁰	−58⁰ to −8⁰
Crouched walk	1	−49⁰ to 35⁰	28⁰ to 54⁰	−33⁰ to 27⁰	−102⁰ to −47⁰	−16⁰ to −6⁰	−61⁰ to −6⁰
Bended walk	1	−29⁰ to 43⁰	26⁰ to 45⁰	6⁰ to 26⁰	−102⁰ to −63⁰	−46⁰ to 16⁰	−32⁰ to −4⁰
Sprawl v1	13	−69⁰ to 37⁰	72⁰ to 83⁰	−19⁰ to −13⁰	−136⁰ to −31⁰	−24⁰ to 71⁰	−48⁰ to 69⁰
Sprawl v2	9	−68⁰ to 19⁰	77⁰ to 99⁰	−66⁰ to 12⁰	−124⁰ to −89⁰	−17⁰ to 22⁰	−58⁰ to −10⁰
							
*ROM:*		*−69⁰ to 43⁰*	*26⁰ to 99⁰*	*−66⁰ to 27⁰*	*−136⁰ to −31⁰*	*−46⁰ to 71⁰*	*−61⁰ to 69⁰*
*Total:*	*25*	*112⁰*	*73⁰*	*93⁰*	*105⁰*	*117⁰*	*130⁰*

Visual inspection of the video of the bended walk trial indicated that the specimen was walking with a more crouched posture. In comparison to the crouched walk trial, the bended walk behaviour required less hip flexion (−29⁰), but slightly greater hip extension (43⁰) and with greater hip abduction (45⁰) and less LAR (6⁰) to pass around the bend. Greater knee extension (−63⁰) and a greater ROM in the ankle (−46⁰ to 16⁰) and MTP (−32⁰) joints were also used.

### 3.2 Muscle moment arms and limb orientations

**FIGURE 5:**
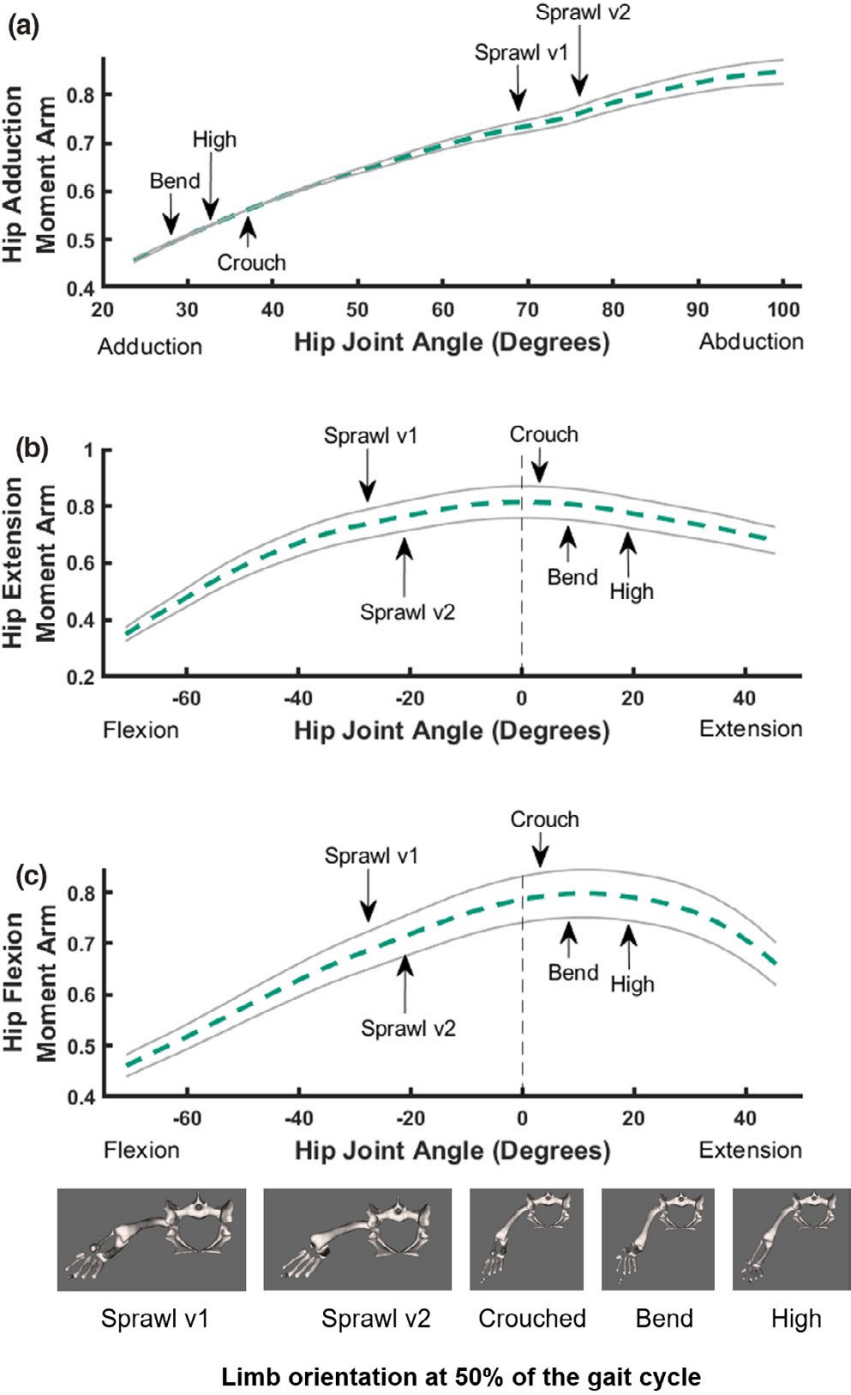
Sum of muscle moment arms for hip abduction (a), extension (b) and flexion (c) normalised by the sum of maximal moment arms, plotted against joint angles for the hip, with representative mid‐stance limb poses for each of the different locomotory behaviours observed in this study indicated (d); in cranial view (right hindlimb shown). The dotted dashed line is the estimated summed moment arm from OpenSim. The solid lines represent the simulated/perturbed error envelopes generated from the 1000 Monte Carlo simulations with up to 20% error incorporated (see Methods)

**FIGURE 6:**
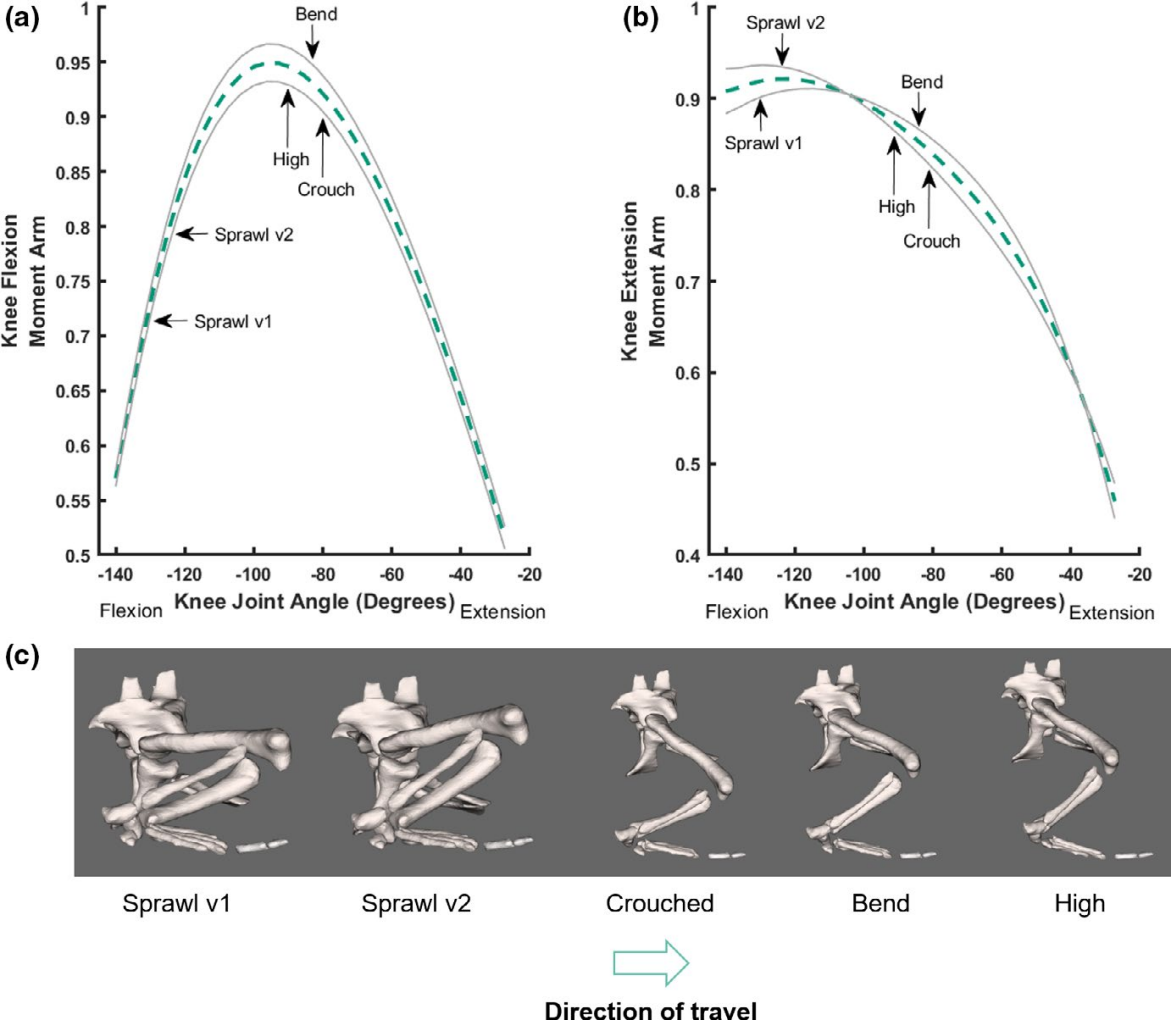
Sum of muscle moment arms for knee flexion (a) and extension (b) normalised by the sum of maximal moment arms, plotted against joint angles for the knee, with representative mid‐stance limb poses for each of the different locomotory behaviours observed in this study indicated, with representative mid‐stance limb poses for each of the different locomotory behaviours observed in this study (c) indicated; in lateral view (right hindlimb shown). Conventions as in Figure 5

**FIGURE 7:**
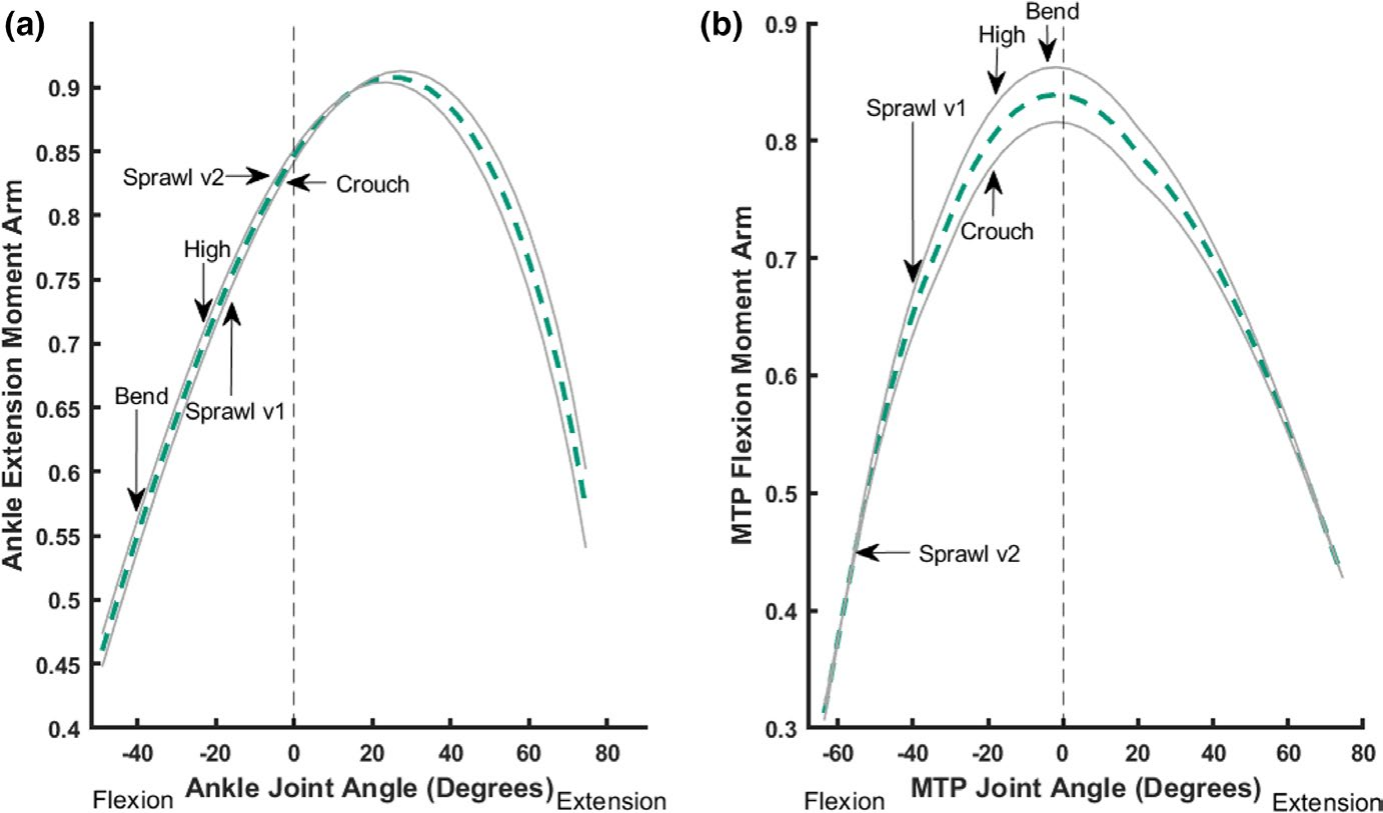
Sum of muscle moment arms for ankle extension (a) and MTP flexion (b) normalised by the sum of maximal moment arms, plotted against joint angles for the ankle (a) and the MTP joint (b). Refer to Figures 5 and 6 for representative mid‐stance limb poses for each of the different locomotory behaviours observed in this trial indicated. Conventions as in Figure 5

We tested whether crocodile hindlimb muscle moment arms are maximised around mid‐stance or are instead optimised during extreme limb positions seen during the sprawls versus the high walks (or neither). Normalised summed muscle moment arms (each muscle's mean across ROM, divided by the summed maximal moment arms) were plotted against joint angles (Figures 5, 6, 7) in which the full ROM per DOF (i.e. the joint angles) used the total ROM presented in Table 5. The summed ±20% Monte Carlo simulations per DOF provided the error margins for each moment arm. Individual muscle moment arms using the original estimated values were plotted against joint angles for each joint in the hindlimb, shown and discussed in Supplementary Information 2. The following observations are pertinent to the mean summed moment arms.

The summed hip adductor moment arms increased almost linearly towards a peak as hip abduction increased (~70⁰ to 80⁰ for sprawling). A sprawling movement with a more retracted femur (i.e. sprawl v2) had a 45% larger summed moment arm than that observed during a high walk. This increase occurred as the pelvis shifted progressively closer towards the ground (i.e. from a high walk to a crouched walk to a sprawl) (Figure 5a,d). The bended walk did not follow this trend, instead exhibiting the lowest adductor moment arm, relatively far from the peak. However, the difference between summed moment arms for the high walk and the bended walk was miniscule (0.69%).

Hip extensor moment arms slightly increased when the distance of the pelvis from the ground increased (i.e. a sprawl vs a crouched walk by 6.6%), but then the extensor moment arms slightly decreased by 5.6% when a high walk was employed and during the bended behaviour, when hip extension was greater than that of more sprawling postures (Figure 5b). A bended walk had a 4.5% larger summed moment arm than that observed during a high walk. The percentage difference between each of the extensor summed moment arms was quite small, with the greatest difference between that of a sprawl and a crouched walk being only 6.6%.

Summed hip flexor moment arms behaved similarly: when the hip was in a more extended position, flexor moment arms were up to 12% greater and reached their peak, whereas when the hip was held in a more flexed position, flexor moment arms decreased (Figure 5c). Whilst the percentage difference between the high, crouched and bended motions were all <2%, the summed flexor moment arms were 11% greater for a high walk than a sprawl, 12% greater for a bended walk than a sprawl and 12% greater for the crouched walk than a sprawl. The difference between summed moment arms for each of the sprawls was only 2.1%.

We compared the poses used during presumed periods of peak limb loading (which would correspond to higher ground reaction forces, near mid‐stance for the five different behaviours) against these summed moment arm patterns (Figure 5). We infer that the hip extensors and flexors have leverages more optimally suited to support or move crouched postures (and to some extent the bended walk too; which was also close to the high walk's values), supporting our first hypothesis. In comparison, the hip adductors are more optimally suited to support sprawling postures, which is not unexpected because the hip is held in a more abducted orientation during a sprawl, incurring greater abductor moments to be resisted (e.g. Blob & Biewener, 2001; Hutchinson & Gatesy, 2000). Considering the error margins from the Monte Carlo simulations, the above observations generally are upheld. This is especially true for hip adduction, in which the more ‘erect’ postures (high, crouch and bended walks) are distinct from the sprawls. Differentiating between peak moment arms in the flexors and extensors is not so straightforward considering the variability within the error margins. As such, the peak moment arm for each of the postures could in fact be the same, or with very little variability between them.

We focused on the flexion–extension axis in the distal joints, starting with the knee (Figure 6). The summed (mean) knee flexor moment arms peaked at moderate knee flexion angles (~−80⁰ to −95⁰), which were adopted during the high, crouched and bended walk behaviours, which were 18%, 17% and 16% greater than the sprawl v2 motion (Figure 6a). Knee flexor moment arms were lower when there were higher knee flexion angles (~‐121⁰ to −130⁰), as present in the sprawling postures. The difference between summed moment arms for the high walk, crouched walk and the bended walk was minor (<2%); or non‐existent considering the Monte Carlo‐simulated error margins; but the difference remained dramatic for those vs. the sprawls.

Summed (mean) knee extensor moment arms behaved differently. Extensor moment arms peaked for the sprawling postures when the knee was in greater flexion and then decreased by up to 10% for the other behaviours (Figure 6b). Knee extensor moment arms were 6.3% greater for the high walk than those of the crouched and bended walks. Considering the error margins from the Monte Carlo simulations, then once again our conclusions from the knee flexor and extensor moment arms are upheld. For both moment arm patterns, a stark difference is evident between the erect‐type postures (especially the crouched walk for knee extension) and the sprawls.

Summed ankle extensor (i.e. plantarflexor) moment arms peaked in more extended ankle positions, but were still distant from optimal values (Figure 7a). There was no obvious pattern identified for the summed moment arms in more sprawling vs. erect postures. The bended walk was the obvious outlier, with a 39% lower summed moment arm than the crouched walk. A high walk had a 17% lower moment arm than that of a crouched walk, whereas the difference between the sprawling trials was 6.7%. The crouched walk and sprawl v2 were the most similar motions, with just 1.1% difference in summed moment arms. Considering the Monte Carlo‐simulated error margins, there were no differences between the sprawl v2 and crouched postures, but the other three behaviours remained distinct from these two and the bended walk retained the lowest summed moment arm.

The summed (plantar) flexor moment arm for the MTP joint increased linearly towards a peak as MTP flexion decreased (Figure 7b). The bended walk exhibited the greatest MTP flexor moment arm (approximating an optimum; very close to the high walk's value as well). In contrast, the sprawling behaviours had the greatest amount of MTP plantarflexion (~−45⁰ to −58⁰), exhibiting the smallest summed MTP flexor moment arms and being 42% (for sprawl v2) and 22% (for sprawl v1) less than the bended walk's peak value.

When the poses used during periods of assumed peak limb loading were compared against these summed moment arm patterns (Figure 7b), we found that the MTP flexors were most optimised to support the bended behaviour, closely followed by the high and crouched walks. We thus infer that the MTP flexor leverages, as modelled here in a relatively simple form, are optimised for more erect postures and perhaps some turning behaviours. Considering the error margins from the Monte Carlo simulations, our conclusions are broadly upheld; especially that the two sprawls had different MTP flexor moment arms from the other three behaviours, which were clustered near the optimum value.

### 3.3 MTU moments

**FIGURE 8:**
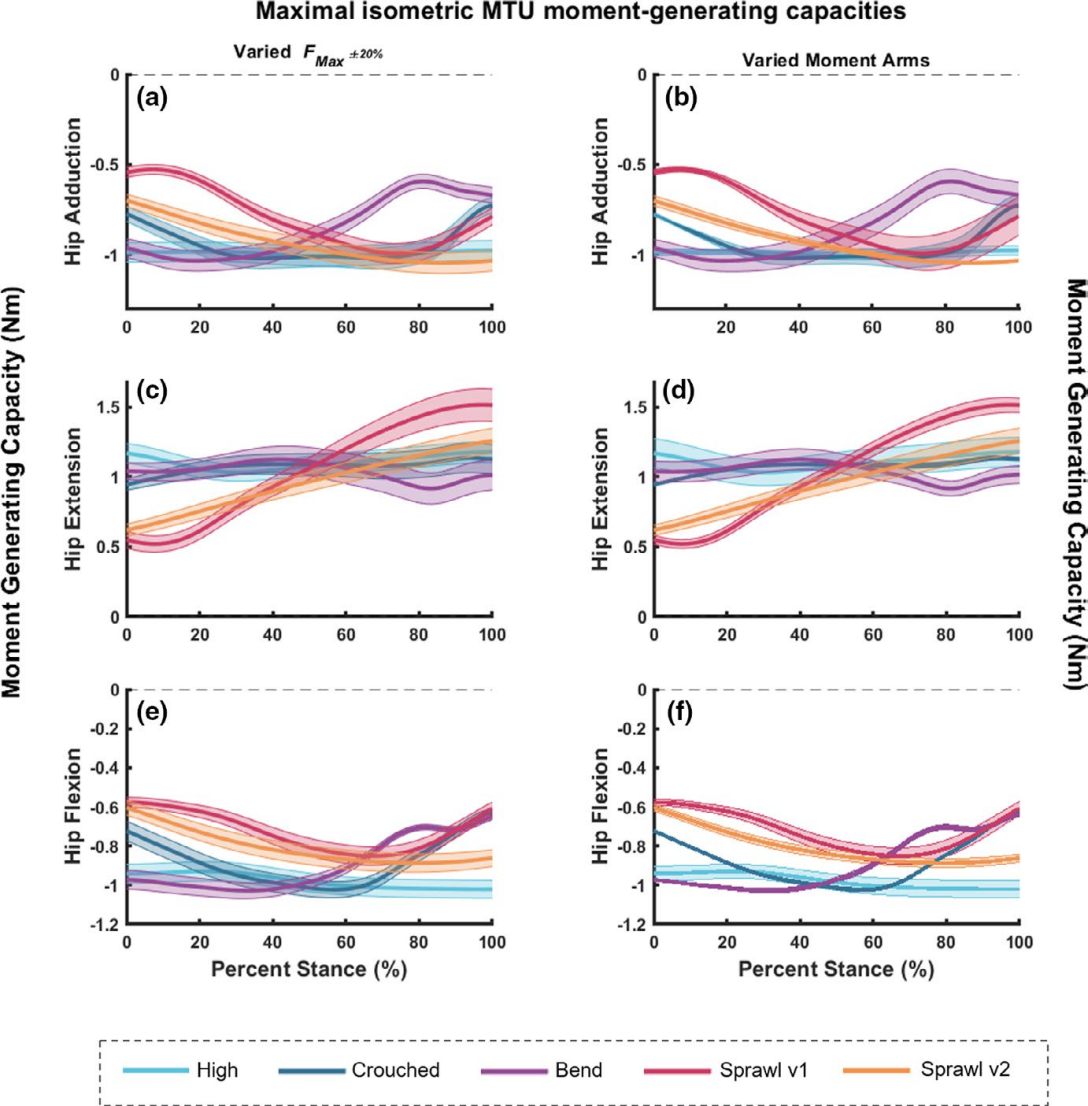
Maximal isometric MTU moment‐generating capacities about the hip joint (a and b for adduction, c and d for extension, and e and f for flexion) for all motions included in this study; during stance phase only. Graphs on the left (a, c and e) represent the estimated moment values from OpenSim with the simulated error margins using $F_{max{\;}^{\pm 20\%}}$ (see Methods). Graphs on the right (b, d and f) also represent the estimated moment values from OpenSim, but instead with the simulated error margins (up to 20% error) using the moment arms varied in the Monte Carlo analysis, which were then used to calculate the isometric moments

**FIGURE 9:**
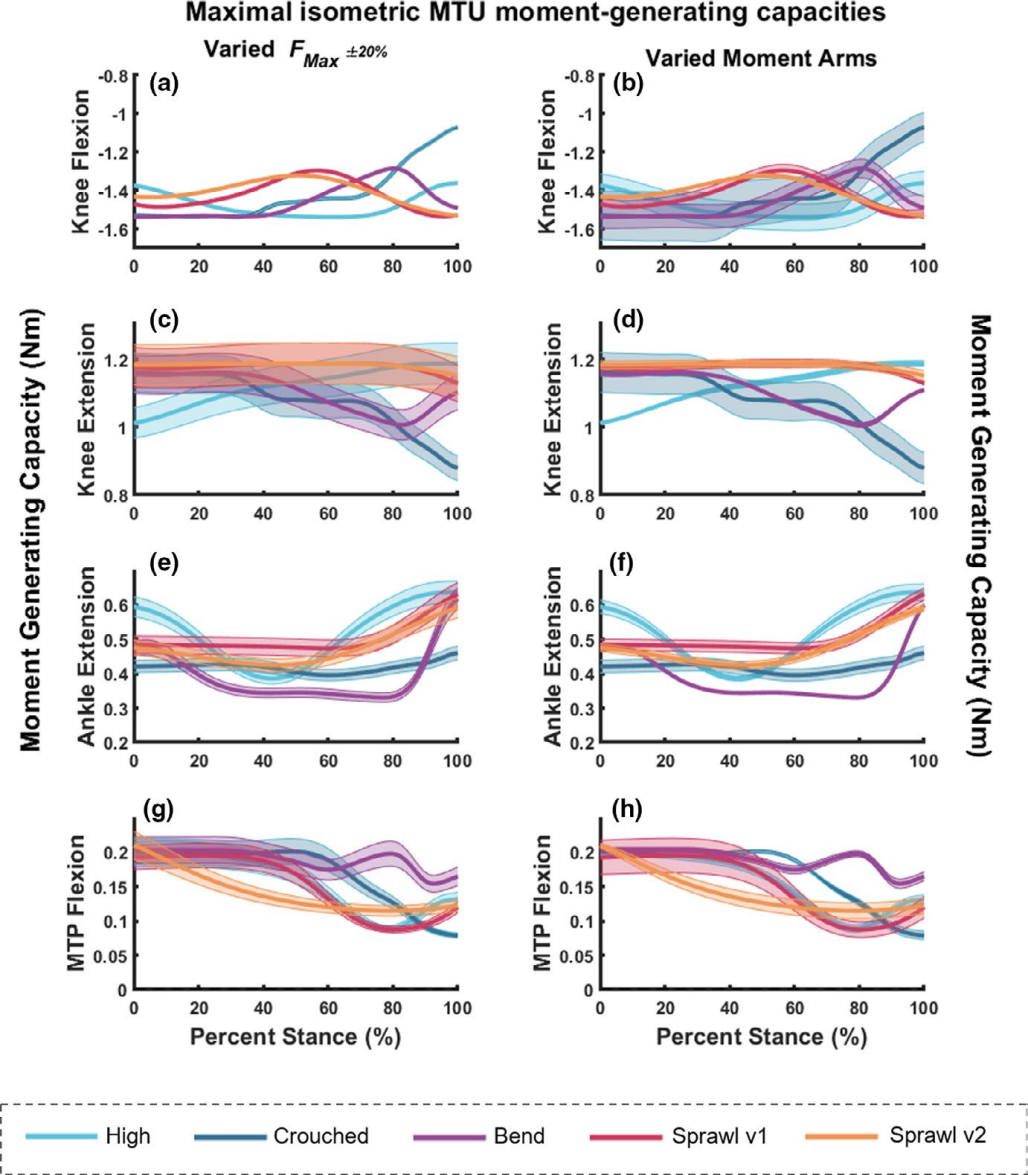
Maximal isometric MTU moment‐generating capacities about the knee (a and b for flexion and c and d for extension), ankle extensors (e and f) and MTP flexors (g and h) for the five behaviours during stance only. Graphs on the left (a, c, e and g) represent the estimated moment values from OpenSim with the simulated error margins using $F_{max{\;}^{\pm 20\%}}$ (see Methods). Graphs on the right (b, d, f and h) also represent the estimated moment values from OpenSim, but instead with the simulated error (up to 20% error) using the moment arms varied in the Monte Carlo analysis, which were then used to calculate the isometric moments

To estimate how the capacity to generate muscular moments varied with limb posture, we calculated the maximal static, isometric muscular moments for each of the hindlimb joints (hip, knee, ankle and MTP) during stance only, for each of the five motions included in this study. We found that the maximal moment curves changed throughout stance for each of the motions (Figures 8 and 9). Peak capacity (most negative moment value) around the hip adduction axis for the hip adductors was optimised around mid‐ to late stance for both sprawling postures (Figure 8a,b). There was a general trend for reduced capacity in early stance for all motions (except for the high and bended walks) and reduced capacity again in terminal stance for most. In contrast, capacity for the bended walk decreased from mid‐stance. The high walk's maximal moments did not change in magnitude during stance, with values ~‐1 Nm. These general conclusions are upheld by the sensitivity analysis with Monte Carlo simulations.

The hip extensors’ peak moment‐generating capacity was reached in late stance for the two sprawl motions (Figure 8c,d). The pattern was more complex for the bended walk, exhibiting modest variations in maximal moments, with a decrease in moments just after mid‐stance and then a slight increase towards toe‐off. In contrast, the crouched and high walks showed negligible variability in moment capacity. Monte Carlo simulations reinforced these general conclusions.

Peak moment‐generating capacity for the hip flexors varied widely (Figure 8e,f). Generally, the values were about half those for hip extension; especially in the two sprawls. For sprawls, capacity increased (became more negative) from early stance, peaking near/after mid‐stance. This pattern reversed for sprawl v1 towards late stance, showing a pattern overall similar to the bended and crouched walks. Again, the high walk showed little if any change. Sensitivity analysis with Monte Carlo simulations supported our conclusions.

The following observations are pertinent to the mean summed isometric moment capacities for the knee flexors. Moment‐generating capacities for both sprawling postures peaked (most negative values) during late stance (Figure 9a,b). The maximal moments for the crouched walk were greatest in early stance, steeply declining towards late stance. The peak moments for the high walk were reached around mid‐stance, when they were comparatively greater than those of the other motions. The peak moment capacity for the bended walk was maintained from ~0 to 40% of stance, steeply reversed, then increased again towards late stance, when just the digits had contact with the ground. However, considering the error margins from the Monte Carlo simulations, differentiating between each of these motions becomes more difficult. Using $F_{max{\;}^{\pm 20\%}}$ to estimate the isometric moment capacities, the simulated error margins for knee flexion were very small (Figure 9a). When perturbing each individual moment arm to simulate the error margins, apparent differences between peak moment capacities per motion were obscured except for the late stance decline for the crouched walk and for the sprawl patterns (Figure 9b), indicating that the moment arm variability had a much greater effect on isometric moment capacities than perturbed $F_{max{\;}^{\pm 20\%}}$.

The maximal moment‐generating capacity for the knee extensors during the sprawling postures did not change much in magnitude throughout stance, with perhaps a slight decrease in moments in terminal stance (Figure 9c,d). Note that peak moment capacities were generally less than for knee flexion. The crouched walk's capacity mainly decreased throughout stance. The patterns for the high and bended walks were different. The peak moment capacity for the high walk occurred in late stance after a steady increase. The peak for the bended walk was also in early stance, where after the maximal moments decreased through mid‐stance and then increased in terminal stance. These observational trends were reinforced by both Monte‐Carlo simulations’ error margins, which mainly were larger for $F_{max{\;}^{\pm 20\%}}$.

Peak moment‐generating capacity for the ankle extensors had peaks in late stance for all motions, although early stance also had large values (Figure 9e,f). Peak capacities tended to be half or less those for the hip and knee. Both sprawling postures were consistent in capacity throughout early to mid‐stance and increased in capacity at ~70% stance. The crouched walk followed a similar pattern to the sprawls, but only had a slight increase in capacity from ~85% stance. The high and bended walks followed different patterns. The high walk had greater capacity in early stance, before a reduction at ~40% stance, after which capacity was increased once again to a peak at toe‐off. The bended walk reduced in capacity by ~25% of stance and maintained a consistent moment‐generating capacity until ~80% of stance, after which a peak was reached via a steep increase in capacity during terminal stance. These observational trends were reinforced by both Monte Carlo simulations’ error margins.

The MTP flexors’ peak moment‐generating capacity for all motions was greatest during early stance, before each of the motions exhibited a decrease in capacity (Figure 9g,h). Sprawl v2 exhibited a steady decline in capacity through stance, whereas sprawl v1 and the high walk did not exhibit decreases until ~40% of stance. Capacity once again increased for both the sprawl v1 and high walks at ~80% stance. The crouched walk steadily decreased from ~55% stance. The bended walk's pattern slightly differed. Whilst this motion did exhibit a decrease at ~60% stance, capacity was increased at ~75% before declining once again. These observations were reinforced by both Monte Carlo simulations’ error margins.

## 4 DISCUSSION

Crocodiles are unusual amongst extant quadrupeds because they are capable of habitually changing their limb posture from a high walk to a sprawl, incorporating extreme degrees of hip, knee and ankle flexion/extension to accommodate such a range of postures. We questioned if the moment arms of pelvic and hindlimb muscles are maximised around mid‐stance (coinciding with approximate peak demands of body weight support), or are instead optimised during limb orientations that are found at the ends of the movement continuum spectrum that we studied (e.g. a sprawl vs a high walk). We found that the peak hip adductor moment arms were more suited to support mid‐stance in more sprawling mid‐stance poses, but were not at optimal values. In contrast, the hip flexor and extensor moment arms were maximised to support more extended postures at mid‐stance; close to optimal values. However, the error margins estimated from the Monte Carlo simulations reveal that the hip extensors’ moment arms did not differ greatly between each of the postures (Figure 5), suggesting that hip extensor leverage cannot be claimed to be optimised to support body weight in mid‐stance postures (which we assume to roughly coincide with peak hindlimb loading). Moving distally down the limb, we found that whilst the knee extensor moment arms were maximised at mid‐stance for the sprawling movements, the knee flexor moment arms were instead maximised at mid‐stance for more erect postures, revealing some amount of differential optimisation of MTU leverage for weight support or joint motion in different behaviours (Figure 6). The ankle extensors’ leverages (Figure 7) were greatest at mid‐stance for limb postures that required increased flexion in the ankle (i.e. the sprawling and crouched postures) but was not at optimal values. The MTP flexors appeared to be more optimal for supporting the three more erect hindlimb poses vs. the sprawls.

By adopting more erect (adducted) limb postures, crocodiles might be able to maintain low muscular stresses to maintain locomotory performance, in a pattern analogous to mammals and birds (Biewener, 1989, 1990, 2005; Bishop et al., 2018a; Gatesy & Biewener, 1991). This may explain why more erect limb postures, such as the high walk, tend to be habitually used by crocodiles for prolonged movement (Cott, 1961; Grigg & Krishner, 2015) or for fast bounding and galloping gaits (Renous et al., 2002). In contrast, more sprawling postures are usually employed for shorter bouts of movement (Reilly & Ellias, 1998), which may explain why the mid‐stance postures for the sprawling movements did not have optimal muscle moment arms for the majority of the DOFs. This postural shift is in contrast to varanids—somewhat comparably sized monitor lizards. Muscle masses and cross‐sectional areas, rather than a change in limb posture, have been found to scale with size to mitigate increased stress in varanids (Cieri et al., 2021; Clemente et al., 2011; Dick & Clemente, 2016), although it is unclear if varanids have greater limb adduction in certain behaviours, as found here for Nile crocodiles (also see Gatesy, 1991). Varanid muscles thus scale differently from Crocodylia, in which muscles in general scale closer to isometry (Allen et al., 2014). Here, we observed that when hindlimb flexion was increased, knee flexion experienced the greatest increase in joint ROM (sprawl v1 in Table 5). Perhaps to support this increased flexion, the mean moment arms of antagonistic knee extensors were maximised in these positions, in contrast to more erect postures. This is because when a more flexed knee posture is adopted, the knee extensors should exert greater forces to counter‐act gravitational forces and knee flexor co‐contraction, preventing the knee from collapsing, although more complex loading may actually be involved (Reilly & Blob, 2003).

Therefore, we conclude that the moment arms of pelvic and hindlimb MTUs are not always maximised during mid‐stance (approximately coinciding with peak body weight support) for all behaviours, but are instead adapted to support a range of different motions in the hindlimb (i.e. exhibit functional trade‐offs), which is not unexpected from sampling five behaviours across a continuum. Knee extensors appeared better suited to support and generate sprawling postures where the knee had extreme ROMs and flexion (~‐120⁰), whereas the flexor moment arms were better matched to a more extended knee. Importantly, unlike the hip adductors and extensors, as well as the ankle extensors and MTP flexors, not all muscles with knee flexor moment arms are predominantly active in stance phase in Crocodylia. Electromyographic activity (mostly for *Alligator* high walks) during the stance phase has been measured for the flexor cruris muscles (*flexor tibialis* heads and *puboischiotibialis*) and the *caudofemoralis longus* (some knee flexor forces may be transmitted via its secondary tendon to the lower limb); unlike *iliofibularis* (Cuff et al., 2019; Gatesy, 1997; Reilly et al., 2005). Hence stance phase knee flexor leverage might be less crucial than knee extensor leverage, particularly for *iliofibularis*. Similarly, almost no muscles with hip flexor moment arms are active in stance; *iliotibialis 1* is an exception (see prior references). Nonetheless, we find it unlikely that the pelvic and hindlimb MTU moment arms overall are tightly ‘tuned’ with mid‐stance (and thus body weight support) during hindlimb stance.

Our second question focused on if crocodiles adopt certain hindlimb postures that optimise their capacity to generate isometric muscular moments about each of the hindlimb joints, thus promoting economical force production (e.g. Fujiwara & Hutchinson, 2012). This question was not well supported by our results. Maximal moment‐generating capacities often did not peak near mid‐stance, but instead in early or late stance, which was generally upheld by our sensitivity analyses. We also found that the moment vs. stance phase curves for each of the five motions included in this study followed different patterns, each with different implications for biomechanical constraints or compromises involved with locomotion. This matches previous findings that external joint moments, particularly around the hip and knee, are not maximal at mid‐stance in normal‐speed high walks, but rather during early and late stance phases (Blob & Biewener, 2001).

However, we do conclude that this hypothesis is supported for the hip adductor muscles during the crouched posture; and possibly the two sprawl behaviours. We found that the position of the hip when crouched (and perhaps sprawling) optimises the moment‐generating capacity of these adductor pelvic limb muscles. This was not entirely unexpected because the adductor muscles are typically’antigravity’ muscles and—with the limb in such an abducted/crouched position—greater adductor moments during these postures would support the body's weight during mid‐stance.

Consideration should also be given here to the duty factor. The duty factor for both sprawling type behaviours was quite low (0.47 ± 0.17 for sprawl v1 and 0.36 ± 0.14 for sprawl v2; Table 2) in comparison to the speeds (0.50 m/s; similar to the other trials’ speeds). These lower duty factors, which are certainly some form of running gaits (trots, as determined from the video capture); should have resulted in greater ground reaction forces and thus higher demands on leverages and moments (assuming minimal tail drag), especially considering a lower effective mechanical advantage of the hindlimb. Perhaps this explains why the knee extensor moment‐generating capacities were greater at mid‐stance for sprawling motions than for the other types of motion.

Some trends in moment‐generating capacity curves were apparent, such as the tendency for the high walk's curves to reach greater peaks at different stages of stance than the other motions, but these patterns were not consistent for every DOF. Furthermore, the steepness of many of these curves indicated that the maximal moment‐generating curves (e.g. knee flexors and extensors, ankle extensors; etc.) may have peaked during swing, not stance, because the peak moment was identified near either the beginning or end of stance. As per above, Gatesy (1997) identified that numerous muscles, such as AMB1, were only recruited during swing, not in stance. Our findings suggest that our hypothesis (i.e. that limb orientations during mid‐stance optimise the moment‐generating capacity of hindlimb MTUs) is not supported for all behaviours, muscles or joints during the stance phase. If the swing phase had been included, we may have found that moment‐generating capacity coincided with swing, or even at foot‐strike phases. This remains untested.

We also caution that mainly slow walking and moderate speeds were included here. Future studies exploring other locomotory behaviours in crocodiles might instead find that maximum moment‐generating capacity is instead reached at faster speeds or gaits such as bounding/galloping (Cott, 1961; Hutchinson et al., 2019; Renous et al., 2002; Webb & Gans, 1982) or alternatively in semi‐aquatic/aquatic movements (e.g. Frey, 1982; Seebacher et al., 2003). Because we did not find that estimated peak moment‐generating capacity was clearly optimised around mid‐stance for most of the studied motions, we postulate that the crocodile hindlimb is suited to a greater repertoire of motions than what has been included here. Our inference fits well with other studies on amphibian, avian and mammalian moment and moment arm optimisation, whereby biomechanical optimisation may vary muscle‐by‐muscle or joint‐by‐joint, or even be associated with a multitude of factors, not just limb posture (e.g. Brown et al., 2003; Cox et al., 2019; Grasso et al., 2000; Hutchinson et al., 2015; Lieber & Brown, 1992; Mai & Lieber, 1990; O’Neill et al., 2013). Nevertheless, we have shown here how distinct groups of muscles have different actions and capacities at varying joint orientations, with potential knock‐on effects on bone stresses. These data can also provide the basis for simulating crocodylian and extinct archosaur movement. However, it should be borne in mind that these results could be complicated by the evolutionary history of Crocodylia, in which posture/locomotory behaviour progressed from a less erect ancestral terrestrial archosaurian condition (e.g. Demuth et al., 2020) to pillar‐erect pseudosuchians and on to buttress‐erect crocodylomorph species, before secondarily adapting to semi‐aquatic lifestyles in the ancestors of Crocodylia (e.g. Bonaparte, 1984; Parrish, 1986; Sullivan, 2015).

Our results are broadly comparable to those of Bates et al., (2015) for the hindlimb of *Alligator mississippiensis*, with a few deviations. Our moment arms were much lower (i.e. by >50% less in the summed hip extensors). This difference can be explained by (1) evident differences in subject size (e.g. the *Alligator* femoral length was 0.137 m, whereas the *Crocodylus* femoral length was 0.070 m, a difference of almost twofold); and (2) different JCS/ACS were used, the latter of which likely explains the differences in the abduction/adduction ROM between *Alligator* (−80⁰ to 40⁰) and *Crocodylus* (26⁰ to 99⁰). The patterns of the hip extensor moment arms were very similar, although the flexors’ pattern was quite different, with the summed flexors in *Alligator* greater at moments of extreme flexion and extension, but reduced for intermediate joint angles (Bates et al., 2015).

### 4.1 Limitations

Our modelled wrapping surfaces and via points may have introduced imprecision into our moment arm results. Such assumptions of muscle paths are a known limitation in all musculoskeletal modelling studies (e.g. Brassey et al., 2017; Hutchinson et al., 2005; Hutchinson et al., 2015; Regnault & Pierce, 2018), even with the inclusion of empirical tendon travel data (e.g. Cox et al., 2019; Hicks et al., 2015). Model evaluation with tendon travel data was not possible due to insufficient remaining cadaveric specimens. Importantly, prior studies (e.g. Hutchinson et al., 2015) have expressed concerns that tendon travel experiments have their own potential flaws from disrupting the 3D geometry of MTUs. Such experiments may also poorly represent joint coordinate systems, leading to imprecise consistency between joint angle estimates (and for multi‐articular MTUs) and ‘cross‐talk’ between 3D angles when expressed in one degree of freedom at a time, as on x‐axes of tendon travel plots; or may involve inconsistent tendon travel results due to regional variation in tendon material properties that cause uncontrolled, nonlinear length changes in MTUs. Further research is needed to refine the validity and applicability of tendon travel data, especially for highly mobile (non‐parasagittal) joints such as in crocodile limbs.

It may be that unmodelled passive tissues (connective and skeletal) play an important role in joint support during any of the five behaviours we studied, as previously suggested by Rankin et al., (2016) for bipedal ostrich locomotion. We did not model tensile forces produced by passive stretch in other tissues, such as ligaments and inside MTUs (Zajac, 1989). However, this is a common issue in biomechanical models; not unique to our study (e.g. Delp & Loan, 2000; Hutchinson et al., 2015). More complex, dynamic simulations would be needed to assess such passive support (e.g. Arnold et al., 2013).

Importantly, the simulated error curves did not change our fundamental results. Our conclusions were upheld with the inclusion of the Monte Carlo simulations in which we addressed moment arm variation, as well as the resulting maximal moment estimation by permitting F_max_ and each muscle's moment arm to also vary by ±20%. Therefore, we argue that our results should compare favourably to precise tendon travel data, because we have estimated potential error informed by trends in available literature data.

### 4.2 Conclusion

Whilst we found that (1) the hip adductors and knee extensors had the greatest moment arms in more sprawling behaviours and (2) more erect postures typically had greater peak moment arms in the hip flexors and extensors, knee flexors and MTP flexors, our results do not well support the hypothesis that biomechanically optimal poses (in terms of leverage as represented here) are adopted during different locomotory behaviours in the Nile crocodiles. Furthermore, isometric moment‐generating capacities broadly seem more complex than being optimised around mid‐stance. Therefore, we infer that the wide range of locomotor behaviours used by this crocodylian species on land prohibits simple optimisation of muscle leverage to any single particular behaviour. Future studies may wish to incorporate more complex tibiofibular or intra‐pedal DOFs and/or to model muscular heads individually (e.g. following the approach developed by Modenese & Kohout, 2020). Nevertheless, our 3D musculoskeletal model has the potential to be used to (1) estimate muscle forces, force‐length changes and moments in extant Nile crocodiles (e.g. via biomechanical simulations of in vivo behaviour); and (2) explore the locomotory capabilities of extinct Archosauria.

## CONFLICTS OF INTEREST

None.

## AUTHOR CONTRIBUTIONS

JRH conceived the study with ALAW. ARC, KBM and JRH planned and collected all experimental and dissection data. ALAW conducted the data analysis assisted by PJB and JRH. OED created the rig used to rotoscope the bones and wrote Supplementary Information 1. ALAW built the model assisted by PJB with JRH. ALAW and OED prepared Figures for the manuscript. ALAW wrote the manuscript aided by JRH. All authors contributed to reviewing the manuscript and approved the final draft.

## Supporting information

Fig S1

Fig S2

Supplementary Material

Video S1

## Data Availability

The calibration images and X‐ray videos used in this study are available on the X‐ray Motion Analysis Research Portal (xmaportal.org, Study Identifier XMA3: Nile Crocodile Hindlimb Study). The Monte Carlo simulation code is available on Figshare: https://figshare.com/articles/software/Monte_Carlo_simulation_code_Matlab_/14216138
