# Supplementary figures and images for: Musculoskeletal modelling of the Nile crocodile (Crocodylus niloticus) hindlimb: Effects of limb posture on leverage during terrestrial locomotion
Source: J Anat. 2021 Mar 23;239(2):424–44. doi: 10.1111/joa.13431 (PMC8273584; doi:10.1111/joa.13431)

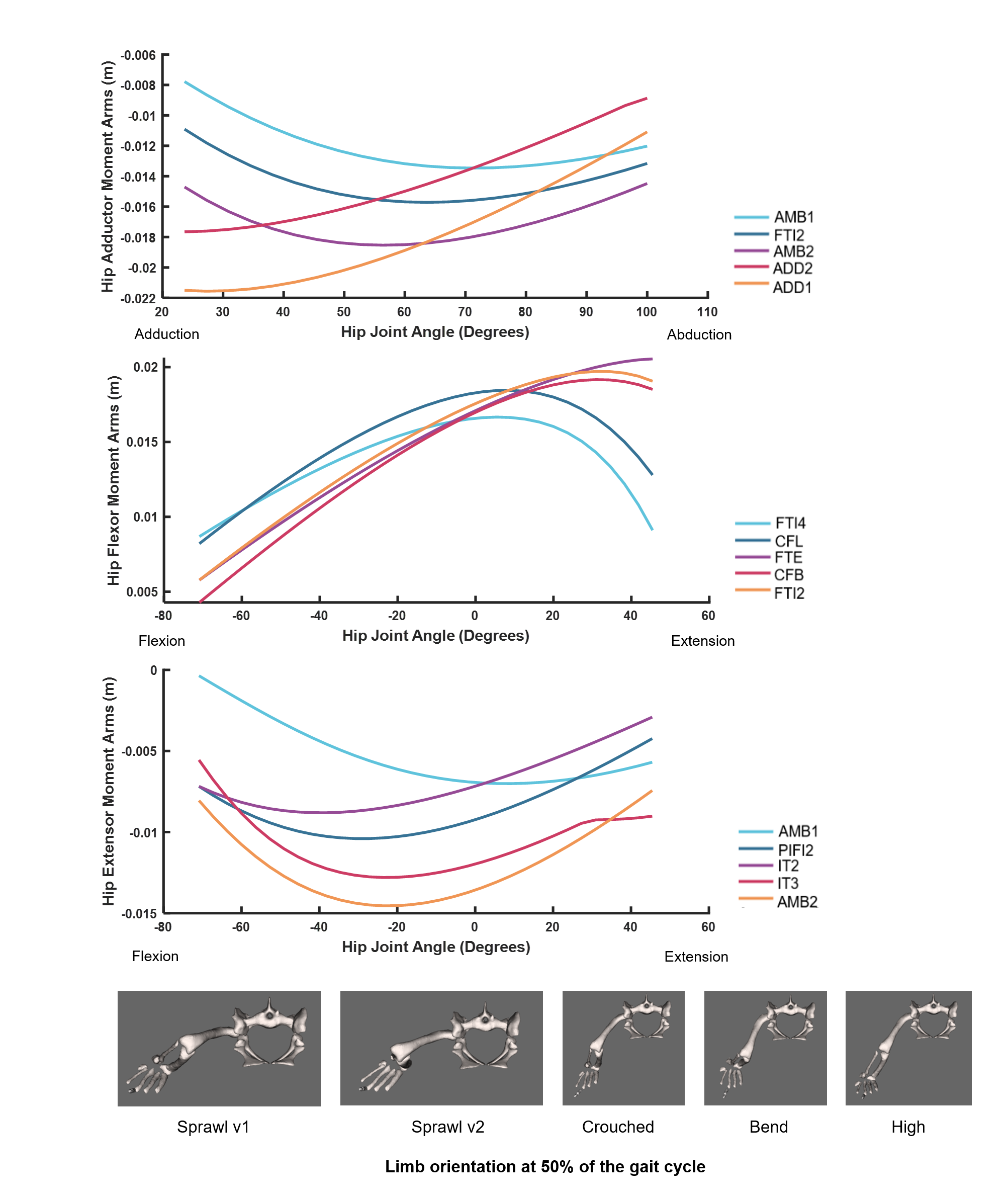

Supplement: Supplementary file 1 — Fig S1 [file JOA-239-424-s004.tif]

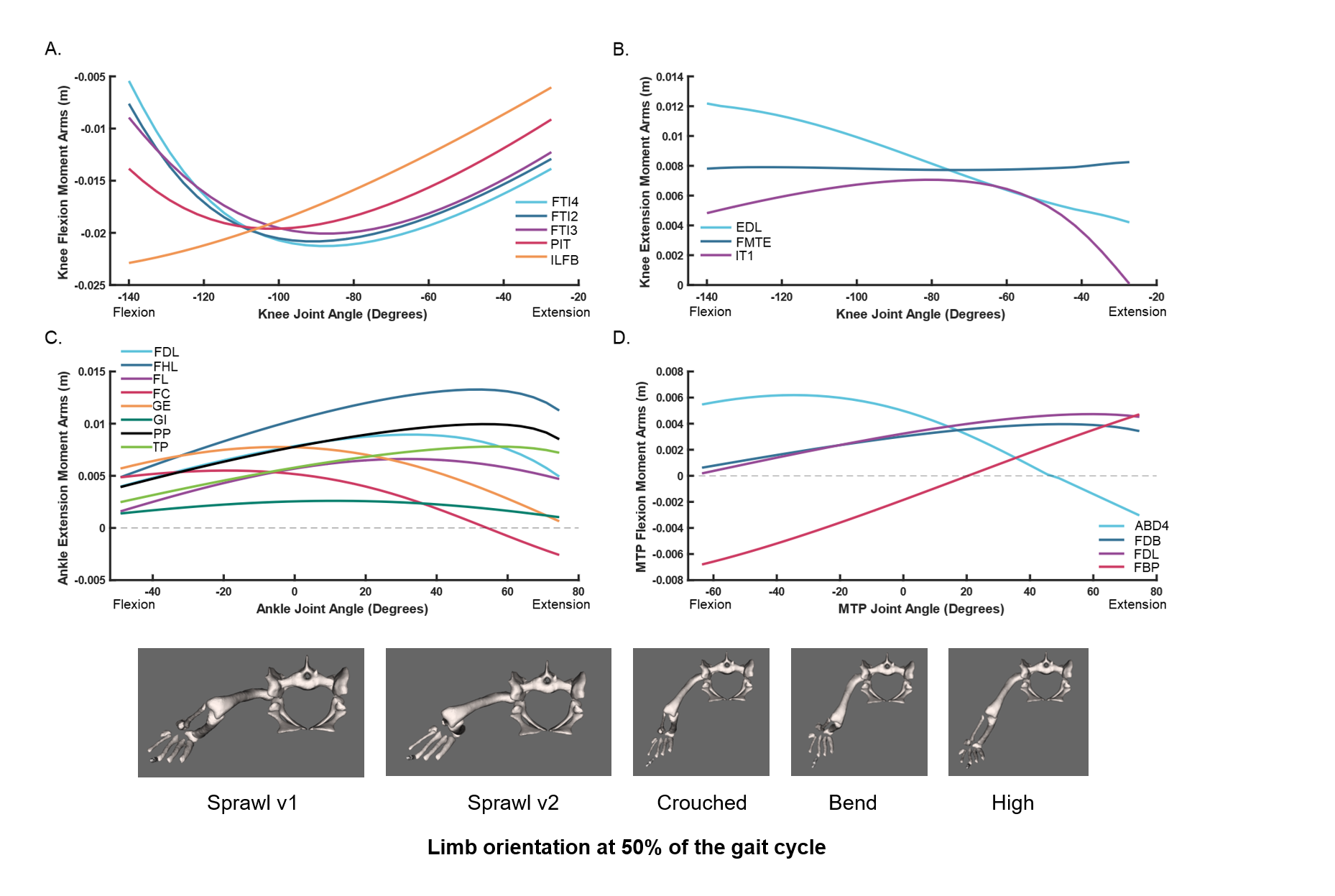

Supplement: Supplementary file 2 — Fig S2 [file JOA-239-424-s001.tif]
