# Supplementary material for: Musculoskeletal modelling of the Nile crocodile (Crocodylus niloticus) hindlimb: Effects of limb posture on leverage during terrestrial locomotion
Source: J Anat. 2021 Mar 23;239(2):424–44. doi: 10.1111/joa.13431 (PMC8273584; doi:10.1111/joa.13431)
Supplement: Supplementary file 3 — Supplementary Material [file JOA-239-424-s003.docx]

**Musculoskeletal modelling of the Nile crocodile (*Crocodylus niloticus*) hindlimb: effects of limb posture on leverage during terrestrial locomotion: Supplementary Information 2**

A.L.A. Wiseman^1*^, P.J. Bishop^1,2,3^, O.E. Demuth^1,4^, A.R. Cuff^1,5^, K.B. Michel^1^, and J.R. Hutchinson^1^.

^1^Structure and Motion Laboratory, Comparative Biomedical Sciences, Royal Veterinary College, Hatfield, United Kingdom.

^2^Geosciences Program, Queensland Museum, Brisbane, Australia

^3^Department of Organismic and Evolutionary Biology, Harvard University, Cambridge, USA.

^4^Department of Earth Sciences, University of Cambridge, Cambridge, United Kingdom

^5^Hull York Medical School, University of York, York, United Kingdom.

Email: [alwiseman@rvc.ac.uk](mailto:alwiseman@rvc.ac.uk)

**Supplementary Information 1**

Detailed instruction for the Inverse Kinematic Constraint rig setup in Autodesk Maya 2019

*Maya joint marionette creation.*

After the anatomical and joint coordinate systems (ACS and JCS, respectively) have been established (see Kambic et al. 2014; Bishop et al. 2020a), a hierarchical joint ‘marionette’ (forward kinematic rig) was created (Gatesy et al. 2010; Arnold et al. 2014) based on the location and direction of the ACSs. The hierarchical order of the forward kinematic (FK) rig was as follows: Pelvis > Hip > Knee > Ankle > MTP > Toe_Tip. The limb was straightened out into the reference pose with a fully extended limb pointing ventrally (Hutchinson et al. 2005), for which the joint Orient of the hierarchical joints was set to (0/0/0) > (90/-90/0) > (180/0/0) > (0/0/0) > (180/0/0) > (0/0/0) and all joint rotations to 0 (see: IK rig set up section below for further details). The *joint Orient* settings enabled the desired joint behaviour and joint rotations to follow the convention of a right-hand rule for counter-clockwise positive rotation (e.g. Sullivan 2007; Kambic et al. 2014). The rotation of the knee, ankle and MTP joint were limited to only allow rotation around their Z-axis (flexion/extension), in line with previous studies (e.g. Hutchinson et al. 2015; Bishop et al. 2020a). The Rotate Order of all joints was set to the Maya default of X-Y-Z (see: Kambic et al. 2014)—this rotation order is equivalent to the Z-Y-X rotation order in OpenSim.

To establish the correct relative spatial position of the implanted beads used for XROMM tracking to the Maya joints in the reference pose, the relevant bones and beads were re-imported from the CT scan dataset (i.e. pelvis with the pelvis beads, tibia and fibula with the shank beads). The beads were temporarily hierarchically parented underneath the bones, which were then brought into position for the extended limb posture using a parent constraint to the previously straightened limb joints, thus ensuring that the relative position of the beads to the bones from the CT scans match up in the now straightened limb. The beads were then unparented and the duplicate bones with their parent constraints deleted. The global position of each bead in this specific position in the world coordinate system thus represented the fully straightened limb in the reference pose. To save the position of the beads and the reference pose for the inverse kinematic (IK) rig (see below) their position was keyed at frame 0.

*IK rig set up.*

To transform the FK rig into an IK rig, an *IK Handle* with a Rotate-Plane Solver was created from the hip joint to the ankle joint. To help the *IK Handle* to recognise which way the knee flexes, the joint Orient was changed to 0.02⁰ (the lowest possible value recognisable by the software during rig creation; the value is negligible) for the Z-axis in the knee joint, which has no effect on the results but is a technical necessity in the set-up. Otherwise, the IK handle was unable to determine the direction of movement.

Three helper locators were created to translate the movements of the beads into the skeletal movement of the crocodiles. The first locator, named Pelvis_Orient_LOC, was used to drive the position and orientation of the pelvis joint. This locator was point constrained to all three pelvis beads (right cranial pelvis, right caudal pelvis and left pelvis) with a weighting of 0.3333 for each bead, thus ensuring that the Pelvis_Orient_LOC was always positioned in the virtual centroid of the triangle between all three pelvis markers. It was then aimed at the right cranial pelvis marker with an *aim vector* of (1/0/0) and an *up vector* of (0/1/0) with the right caudal pelvis marker as *World Up Object* for the *Object Up World Up Type*. This ensured that the orientation of the Pelvis_Orient_LOC was always in relation to a virtual plane through the three pelvis beads.

The same procedure was used to create the Shank_Marker_Centroid_LOC. It was point constrained to the shank beads (proximal tibia, distal tibia and fibula) with a weighting of 0.3333 each. It was then aimed at the distal tibia bead with an *aim vector* of (1/0/0) and an *up vector* of (0/1/0) with the distal tibia marker as *World Up Object* for the *Object Up World Up Type*.

A third locator, named Knee_AIM_LOC, was created to drive the rotation of the IK Handle to bring the knee joint in line with the shank beads. First, it was brought into the exact position of the Shank_Marker_Centroid_Loc and then moved cranially by an arbitrary amount (2cm in our case). It was then moved laterally to bring it in line with the XY plane of the knee ACS to counteract the mediolateral offset of the Shank_Marker_Centroid_LOC. This locator was then hierarchically parented underneath the Shank_Marker_Centroid_LOC. This ensured that the position and orientation of the Knee_AIM_LOC were driven by the shank beads and that it was always positioned on a plane through the hip, knee and ankle joint, meaning that knee flexion angles did not influence the relative position of the Knee_AIM_LOC. The previously created *IK Handle* was then constrained to the Shank_Centroid_LOC using a *parent constraint* and to the Knee_AIM_LOC using a *pole vector constraint.*

*Implications*.

This IK setup enabled us to translate the bead motions into the kinematic movement of the crocodile hindlimb matching up with the X-ray shadows of the XROMM data. The position and orientation of the pelvis were driven by the pelvis beads, while the orientation and position of the crus were controlled by the shank beads. The orientation of the femur was driven by the relative position and orientation of the crus to the pelvis. The desired long-axis rotation of the femur was guaranteed as the Z-axis of the distal femur ACS was forced to be perpendicular to the shank long-axis by the rig setup.

The ankle required additional long axis rotation to ensure that the pes segment lay flat on the ground, rather than penetrating the ground. This DOF was rotated to -35º for all rigs across all motions (i.e. the high, crouched, bended walks and sprawl movements). This rotation in the ankle joint was static throughout each motion. Only flexion-extension at the ankle was permitted to vary throughout each motion. More invasive surgeries would have been required to obtain sufficient information on 3D pedal bone motions to represent the complex motions of these joints more realistically, but we felt we adequately captured the fundamental kinematics.

**Supplementary Information 2**

*Muscle moment arms*

Individual muscle moment arms were plotted against joint angles for each joint in the lower limb. For simpler visualisation, rather than plotting all 47 muscles (these data are summarised in the summed moment arm graphs in Figures 5-7), we plotted the muscles which had the greatest moment arm peak for each of the orientations and discuss only the major findings. Interestingly, we found that as the hip became more abducted (i.e., the hip was more abducted during stance when more sprawling versus in a high walk), some major muscles antagonistic to abduction (e.g., ADD1 and ADD2) decreased in moment arm; becoming less negative in values (Figure S1a). ADD2 had the greatest peak moment arm at greater hip adduction, corresponding to the bended and high walks in which the hip had the most adduction. The ADD1 muscle’s moment arm followed the same pattern along the adduction-abduction axis as observed for *Alligator* (Bates et al. 2015). However, the AMB1, AMB2 and FTI2 muscles had a greater adduction moment arm during more sprawling postures (i.e., when the hip was more abducted). The pattern for these muscles explains the predominant pattern observed in Figure 5a.

All hip extensors increased in moment arm as hip extension increased until ~5⁰, after which the FTI4 and the CFL moment arms declined as extension increased further (Figure S1b). The CFB and FTI2 moment arms reached a plateau just before maximal extension, whilst the FTE moment arm continued to increase with greater hip extension. When the poses that crocodiles used during mid-stance were compared against these patterns, we found that all peak moment arms increased when comparing a sprawl versus a crouched walk, following the trend in Figure 5b. Peak moment arms for the FTI2 slightly matched the high walk posture.

The hip flexors tell a different story (Figure S1c), but generally reflecting the trend in Figure 5c. AMB1’s moment arm increased (more negative values) before eventually plateauing (at ~0⁰) as hip extension increased, although this muscle’s moment arm was quite low, reaching a peak of just -0.007m. Gatesy (1997) reported that the AMB1 muscle was only active during swing, whereas in this study we found minimal moment arms during stance in our musculoskeletal model. We predict that if the swing phase were to be included here then we would see the peak moment arm for this muscle during swing, congruent with previous studies of muscle activity. In contrast, the PIFI2 and IT2 moment arms decreased as extension increased past ~-40⁰ to -20⁰ hip flexion. The peak moment arms for the AMB2 and IT3 muscles were reached in their plateaus ~-20⁰. When the limb became more erect into a crouched or high walk (bended walk included here), the peak hip flexor moment arms for the PIFI2, IT2, IT3 and AMB2 were less than those for the sprawling postures. The CFB, CFL and PIFI2 muscles’ moment arms followed the same patterns along the extension-flexion axis as modelled for *Alligator* (Bates et al. 2015).

We found for most knee flexor muscles (FTI2, FTI3, FTI4 and PIT), the peak moment arm (most negative) was reached during movements which involved a more extended knee posture ~-80 to -100⁰ flexion – the high and crouched walks (Figure S2a); matching Figure 6a. The peak moment arms for these muscles were reduced in comparison for more sprawling postures. The ILFB muscle behaved somewhat differently: its moment arm increased throughout the knee ROM as knee flexion increased to extreme values (~-140⁰), meaning that the ILFB was not well suited to support or move mid-stance postures with highly extended knees, in terms of simple leverage. Following Gatesy (1997), the ILFB is active around the stance-swing transition during a high walk (also see Cuff et al., 2019), so its moment arm may be more related to swing phase dynamics (e.g., driving marked knee flexion).

The knee extensor moment arms exhibited a different pattern (Figure S2b). The IT1 moment arm slightly increased as knee extension increased, reaching a peak at ~-70⁰ before the moment arm decreased once more. The FMTE moment arm remained almost static throughout the knee’s full ROM. When the poses that crocodiles used during periods of peak limb loading were compared against these patterns, we inferred that the IT1 muscle was more optimally suited (at the knee) to support or generate the crouched, high and bended walks, whereas the FMTE was more difficult to associate with a particular behaviour. The EDL’s role in knee extension is seldom considered but it had a large extensor moment arm in flexed knee poses, declining steeply by about 50% with extension. The complex patterns for these muscles, similar to those for the few other extensors (IT2, IT3, AMB1, AMB2 and FMTI), still broadly reflected the trend in Figure 6b.

Almost all ankle muscle moment arms (Figure S2c) had more consistent actions than those of the knee muscles. The muscle moment arms generally increased with ankle extension, usually peaking ~40⁰ to 60⁰ of extension. Most muscles followed the pattern of increasing moment arm as ankle extension increased until ~60⁰. These peak values are far from the ankle poses used around mid-stance, as per Figure 7a. Two exceptions were the GE and FC muscles, which both peaked at more neutral postures (~-10⁰ to 0⁰), closer to values used near mid-stance in some behaviours. Interestingly, the FC muscle switched at 55⁰ extension to act antagonistically against the extensors.

The peak moment arms for the FDB and FDL muscles acting around the MTP joint steadily increased as the MTP joint adopted more extended postures (grossly following Fig. 7b), with peak flexor moment arms at positions of extreme extension (e.g., ~60⁰), thus providing better antigravity support. The ABD4 and FBP muscles had unique patterns. ABD4 had greater moment arms during flexed positions (~-40⁰ to 20⁰), after which the muscle changed to act antagonistically against the flexors during highly extended postures (>40⁰). In contrast, the FBP first acted as an MTP extensor during flexed positions, after which the muscle switched at ~20⁰ MTP extension to act as a flexor. It is hard to establish whether these switches of actions are biologically representative, or are instead artefacts of modelling simplifications in the foot. Other artefacts of simplification were found in the action of the DE4 muscle. This muscle is in the dorsal muscle compartment of the foot and should be acting as an extensor of the MTP joint. Rather, we found that this muscle was acting as a flexor in contrast to the other digital extensors (DE1-3). We deemed this a spurious result of simplistic modelling of the foot rather than a biological action of the DE4 and so have omitted details of the DE4 here.
